# Assessing oxime reactivation efficacy using principal component analysis: Insights from nerve agents inhibited human butyrylcholinesterase

**DOI:** 10.5599/admet.3361

**Published:** 2026-05-28

**Authors:** Goran Šinko, Tena Čadež, Zrinka Kovarik, Nikolina Maček Hrvat

**Affiliations:** 1 Division of Toxicology, Institute for Medical Research and Occupational Health, Ksaverska cesta 2, HR-10000, Zagreb, Croatia; 2 University of Zagreb, Faculty of Science, Horvatovac 102, HR-10000, Zagreb, Croatia

**Keywords:** Cholinesterase, organophosphate, ADME, asoxime (HI-6), pralidoxime (2-PAM), pesticide

## Abstract

**Background and purpose:**

The toxicity of organophosphorus compounds (OPs) and related nerve agents (NAs) impairs the cholinergic system via irreversible inhibition of acetylcholinesterase (AChE) activity by phosphylation of the catalytic serine. Reactivation of the enzyme activity largely depends on the structural compatibility between the enzyme, an oxime reactivator, and the specific OP compound.

**Experimental approach:**

For this study, we used our recently published data on the reactivation of human butyrylcholinesterase inhibited by the NAs sarin, cyclosarin, tabun and VX, using a library of 115 oximes. We compared these results with oximes’ ADME (absorption, distribution, metabolism, and excretion) parameters relevant to central nervous system activity using principal component analysis (PCA). PCA facilitated the examination of these relatively large datasets by increasing interpretability while minimizing information loss.

**Key results:**

Three components with eigenvalues above 1 resulted in 72 % of the cumulative proportion of variance and described 27 variables. PC1 created transformed data that had negative values for most oximes with high reactivation potential, while showing large positive values for oximes with moderate and low efficacy. Distribution of 27 loadings, representing 27 variables, produced a set of 9 positive and 18 negative loadings representing negative and positive data correlation. The efficacy of oxime reactivation was highly correlated with the parameters describing its structure: molecular weight, rotational bonds, molecular volume, and molecular surface area.

**Conclusion:**

A large dataset was efficiently analysed by maximizing the preservation of variability and generating new, uncorrelated variables. To our knowledge, this study is the first to apply PCA to assess oxime’s reactivation efficacy, thus providing insights into the relationships between oxime properties and their efficacy in restoring OP-inhibited cholinesterase activity.

## Introduction

The design of oxime reactivators for both acetylcholinesterase (AChE) and butyrylcholinesterase (BChE) inhibited by organophosphorus compounds (OPs), including nerve agents (NAs), remains challenging, with limited success [1]. The main problem in designing effective cholinesterase (ChE) reactivators is the poor structure-activity relationship of newly developed oximes, which very rarely achieve the reactivation efficacy of standard oximes such as pralidoxime (2-PAM), asoxime (HI-6), obidoxime and dipyroxime (TMB-4) [2]. The structure of the OP that inhibits ChE is another critical factor in the effectiveness of reactivation. For example, oxime HI-6 is a potent reactivator of cyclosarin-inhibited AChE, a moderately effective reactivator of sarin or VX-inhibited AChE, but an ineffective reactivator of tabun-inhibited AChE [3].

Worek and co-workers commented on the structure-activity relationship in the development of oxime reactivators: “Since the invention of the first clinically used oxime, pralidoxime (2-PAM) in the 1950s, ongoing research attempted to identify more effective oximes. In fact, several thousand oximes were synthesized in the past six decades. These include charged and non-charged compounds, mono- and bispyridinium oximes, asymmetric oximes, oximes with different substitutes and more recently non-oxime reactivators. Multiple *in vitro* and *in vivo* studies investigated the potential of oximes to reactivate OP-inhibited AChE. The inconsistent effectiveness of oximes in the treatment of OP-pesticide-poisoned patients led to a continuous discussion on the value of oximes. In order to provide a forward-looking evaluation of the significance of oximes in OP poisoning, multiple aspects, including intrinsic toxicity, *in vitro* reactivation potency, efficacy and pharmacokinetics, as well as the impact of the causative OP, have to be considered.” [1].

Oximes are primarily designed to reactivate AChE, given its vital role in neurotransmission, thereby attenuating toxic OP effects. Yet, the related enzyme BChE, which, unlike AChE, doesn’t have an essential physiological role, has been recognised as a pseudo-catalytic scavenger of OP compounds if paired with an effective BChE oxime reactivator [4-8]. An efficient pseudo-catalytic scavenger would degrade the OP compound before AChE inhibition, thus protecting the victim from the poisoning [4,9]. However, effective AChE reactivators show poor efficacy in BChE reactivation due to differences in the amino acid composition lining the active-site gorge of AChE. The design of effective BChE reactivators needs to be guided by specific structural properties of the BChE active site that arise from differences in the composition of aromatic residues. Instead of the six aromatic residues present in the AChE active site, matching aliphatic residues are found in the BChE active site, thus changing the aromatic properties and enlarging the volume of the BChE active site ~0.200 nm^3^ (200 Å^3^) [10]. As mentioned, reactivation effectiveness greatly depends on the structure of the OP compound that inhibited ChE. Upon inhibition, the volume of the active site is reduced depending on the type of inhibiting OP compound, which is one reason oxime reactivation efficacy varies significantly among different OPs [11,12]. Finding a universal reactivator, the oxime that would be effective in the reactivation of ChE inhibited by various OPs, would be considered the ultimate goal, and *in silico* studies can be a helpful tool.

**Figure 1.**
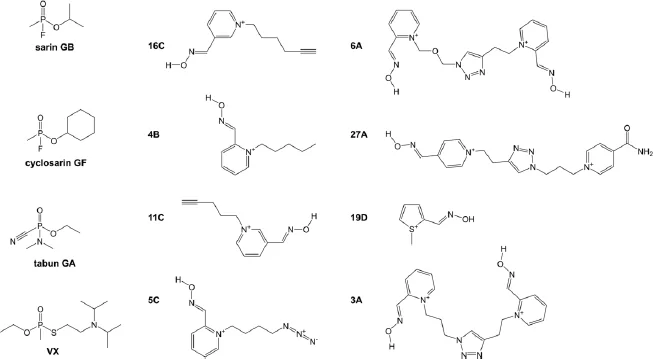
Chemical structures of effective oxime reactivators of BChE inhibited by organophosphorus compounds: tabun (GA), sarin (GB), cyclosarin (GF), and VX, screened from a library of 115 oximes [9].

In this study, we used our previously published results on the *in vitro* evaluation of the kinetic properties of a library of 115 oximes for the reactivation of BChE inhibited by OPs: sarin (GB), cyclosarin (GF), tabun (GA), and VX [9]. The library contained triazole oximes synthesised using the click-chemistry method and their pyridinium or miscellaneous building blocks (Figure 1). A set of standard oximes known for their efficacy in AChE reactivation inhibited by OPs: 2-PAM, HI-6, obidoxime, and TMB-4, was used for comparison. The aim of the study was to verify if the principal component analysis (PCA) could be used as a statistical method for the evaluation of the oxime’s reactivation efficacy. PCA is a technique for analysing relatively large datasets, and it was chosen for its ability to increase interpretability while minimizing information loss [13-16]. We analysed the reactivation efficacy of the library of oximes using kinetic parameters together with pharmacological parameters such as ADME (absorption, distribution, metabolism, and excretion) and drug design parameters. The rationale for this type of analysis is also the prediction of CNS (central nervous system) reactivation activity of the studied oximes. Due to their permanent positive charge, which limits passive diffusion across the blood-brain barrier (BBB), these oximes may have limited reactivation activity toward ChEs in the CNS [17,18].

## Experimental

Kinetics obtained for a library of 115 oximes screened for reactivation of BChE inhibited by the NAs tabun, sarin, cyclosarin and VX were adopted from the study by Čadež *et al.* [9] Dataset determined for each NA contained reactivation parameters obtained with 0.1 mM oximes: the first-order observed reactivation rate constant (*k*_obs_ / min^-1^), the maximal percentage of reactivation (React_max_; %), time at which React_max_ was achieved (*t* / h), and BChE inhibition, % by the 0.01 mM oxime calculated against control enzyme activity (Supplementary material, Table S1). Due to a large span of *k*_obs_ values (four orders of magnitude), normalisation was applied to narrow it, which is beneficial for the PC analysis.

### ADME dataset of selected oximes

The 3D structures of a library of 111 oximes and a set of four standard oximes were minimized with the MMFF94 force field using ChemBio3D Ultra 12.0 (PerkinElmer, Inc., Waltham, MA, USA). The Discovery Studio 21.1 (BioVia, San Diego, CA, USA; https://www.3ds.com/products/biovia/discovery-studio) ADME (absorption, distribution, metabolism, and excretion) descriptor protocol was used for the calculation of the following molecular parameters: lipophilicity coefficient (Alog *P*98) and topological polar surface area (PSA 2D) [19-21]. The correlation between Alog *P*98 and PSA 2D was used to predict BBB permeability from a training set of known CNS-active compounds with good adsorption and BBB permeability properties. In addition to parameter Alog *P*98 and PSA 2D, physicochemical properties: molecular weight (MW), number of hydrogen bond donor atoms (HBD), and number of hydrogen bond acceptor atoms (HBA), dipole moment, molecular surface, and fraction of polar molecular surface were calculated [22].

### Principal component analysis (PCA) of the oxime library

The reactivation dataset of the studied oxime library in combination with the ADME dataset was analysed using principal component analysis (PCA) statistical model in GraphPad Prism 9 software (Dotmatics, UK; https://www.graphpad.com/features). The first PCA model, applied to all four OP compounds and including 27 variables in total, generated 27 PCs with matching variances and eigenvectors of the covariance matrix. The second PCA model was applied to each of the four OP compounds, with 18 variables in total, generating 18 PCs with matching variances and eigenvectors. For each variable, the loading was calculated as the Pearson correlation coefficient between the original data and the principal component scores [13,14]. Loadings were calculated by scaling the eigenvectors (weights) by the square root of their corresponding eigenvalues, representing the contribution of a variable to a principal component. Plots for PCA visualization comprise PC1 *vs.* PC2, and PC2 *vs.* PC3 correlation plot, and a plot of PC1-PC3 loadings distribution. Prior to analysis, data describing first-order observed reactivation rate constant (*k*_obs_ / min^-1^) were normalised, calculating a negative logarithmic value due to a very broad data range, which is four orders of magnitude. Reactivation of inactive compounds resulted in *k*_obs_ equal to zero, and due to logarithmic normalisation, a negative logarithmic value for inactive compounds is set to 5.

## Results

### Analysis of normalised reactivation rate constants

**Figure 2.**
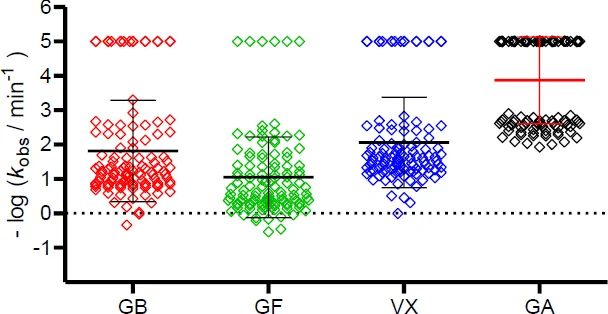
Statistical analysis of the normalised first-order observed reactivation rate constant (*k*_obs_), determined for 115 oximes in reactivation of BChE inhibited with sarin (GB), cyclosarin (GF), tabun (GA) and VX [9]. Due to the logarithmic normalisation, a negative logarithmic value for inactive compounds in BChE reactivation is set to 5 (*k*_obs_ = 0 min^-1^). Black or red bars represent mean ± standard deviation of the normalised *k*_obs_

Initial analysis of the normalised first-order observed reactivation rate constant (*k*_obs_) for 115 compounds tested for BChE reactivation after inhibition by GA, GB, GF, and VX showed that the overall oxime reactivation efficacy followed the order GF > VX > GB > GA. Mean normalised *k*_obs_ values with standard deviation are 1.05±1.17, 1.82±1.45, 2.06±1.32 and 3.87±1.26 for GF, GB, VX and GA reactivation, respectively (Figure 2).

### PC analysis of BChE reactivation efficacy

**Figure 3.**
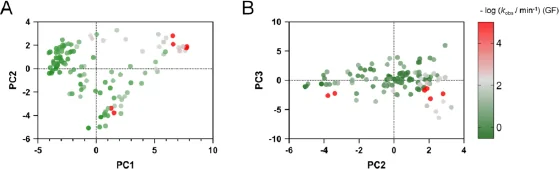
Principal component (PC) analysis of 115 compounds tested for BChE reactivation after inhibition with sarin (GB), cyclosarin (GF), tabun (GA), and VX using a dataset comprising 27 variables. (A) Correlation of PC1 and PC2 containing 42.4 and 15.5 % of proportion of the variance, and (B) correlation of PC2 and PC3 containing 15.5 and 14.1 % of proportion of the variance (Table 1). Dark green circles represent the most active oximes in the BChE reactivation, and dark red circles represent the inactive oximes in the reactivation

**Figure 4.**
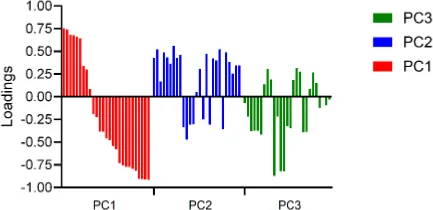
Distribution of principal component (PC) loadings. The dataset includes 27 variables for 115 compounds tested for BChE reactivation after inhibition with sarin (GB), cyclosarin (GF), tabun (GA), and VX. Bars are ranked by the values of PC1 loadings. Top PC1 loadings are the time at which React_max_ was achieved and normalised first-order observed reactivation rate constant (*k*_obs_), for GB, GF and VX. The lowest PC1 loadings are molecular weight, number of rotational bonds, molecular volume, and molecular surface area. For further details about the distribution of variables, please see Table 1

Principal component (PC) analysis of 115 compounds tested for BChE reactivation after inhibition with GA, GB, GF and VX, using a dataset comprising 27 variables, resulted in the selection of three PCs, each with eigenvector values higher than 1, and 72.04 % of cumulative proportion of variance (Table 1, Figure 3, Figure S1). Eigenvector values of the covariance matrix were 11.46, 4.18 and 3.8 for PC1, PC2, and PC3, respectively. Calculated loadings for 27 variables indicate the contribution of each variable to each PC; they are limited to the range ±1. The sign of the loading indicates a positive or negative contribution to the PC, while its magnitude provides a direct measure of the PC linear relationship. Principal component 1 (PC1) contains 42.4 % of the proportion of variance and has 8 positive PC loadings related to reactivation parameters, normalised *k*_obs_, and the time (at which React_max_ was achieved), for all four OP compounds. Oxime inhibition of control enzyme activity, and the maximal percentage of reactivation (React_max_) parameters have negative loading values. Interestingly, all pharmacological parameters also have negative loading values. Molecular weight, number of rotational bonds, molecular volume, and molecular surface area have loadings below 0.9 (Table 1, Figure 4).

**Table 1.** Principal component (PC) analysis of 115 compounds tested for BChE reactivation after inhibition with GA, GB, GF and VX. List of loadings for 27 variables ordered according to PC1. The proportion of variance is listed in the brackets with the cumulative proportion of variance for the selected components 72.0 %

	Loadings		
	PC1 (42.4 %)	PC2 (15.5 %)	PC3 (14.1 %)
Eigenvector value	11.46	4.18	3.82
Time, h (GB)	0.755	0.427	-0.069
-log (*k*_obs_ / min^-1^) (GB)	0.741	0.523	-0.218
-log (*k*_obs_ / min^-1^) (GF)	0.681	0.167	-0.381
-log (*k*_obs_ / min^-1^) (VX)	0.678	0.489	-0.373
Time, h (VX)	0.663	0.433	-0.375
Time, h (GF)	0.643	0.365	-0.416
-log (*k*_obs_ / min^-1^) (GA)	0.338	0.562	0.138
Molecular fractional polar surface area, nm^2^	0.301	0.429	0.304
Time, h (GA)	0.084	0.458	0.189
ADMET Alog *P*98	-0.189	-0.333	-0.870
React_max_, % (GA)	-0.226	-0.470	-0.222
Alog *P*	-0.383	-0.310	-0.819
log *D*	-0.385	-0.304	-0.822
Dipole magnitude	-0.459	0.054	-0.325
Oxime inhibition, %	-0.477	0.306	-0.346
React_max_, % (GF)	-0.542	-0.249	0.184
Number of H bond donor atoms	-0.580	0.470	0.318
React_max_, % (VX)	-0.731	-0.306	0.276
Number of rings	-0.756	0.424	-0.393
Number of aromatic rings	-0.773	0.399	-0.388
Number of H bond acceptor atoms	-0.774	0.523	0.088
React_max_, % (GB)	-0.793	-0.357	0.265
ADMET PSA 2D	-0.814	0.491	0.151
Molecular weight	-0.903	0.382	-0.122
Number of rotational bonds	-0.907	0.255	0.005
Molecular volume, nm^3^	-0.911	0.346	-0.095
Molecular surface area, nm^2^	-0.915	0.343	-0.031

Principal component 2 (PC2) accounts for 15.5 % of the variance and includes 20 positive loadings, encompassing the reactivation parameters and the majority of pharmacological descriptors. The exceptions were log *D*, Alog *P* and ADMET Alog *P*98, which are all related to describing a compound’s lipophilicity *via* the octanol-water partition coefficient. The reactivation parameter React_max_ shows negative loading values for all four OP compounds.

Principal component 3 (PC3) accounts for 14.1 % of the variance and has 10 positive loadings, including React_max_ for GB, GF, and VX, with the exception of GA. The reverse pattern is observed for the normalised *k*_obs_ and the time at which React_max_ was achieved, where positive loadings were attributed to GA and negative loadings to GB, GF and VX.

Distribution of transformed dataset points after correlation of PC1 and PC2 (Figure 3) shows the position of the oxime **16C**, with the highest reactivation efficacy according to normalised GF *k*_obs_, in the lower part of Figure 3A with coordinates (0.4, -4.1), and one of inactive oximes, **19D**, in the far-right part of the figure with coordinates (7.7, 1.8). A distinct grouping of effective oximes can be observed in the top left part of the plot, corresponding to positive PC2 values. The PC3 axis values, in correlation with PC2, appear to discriminate oximes based on their reactivation efficacy, because most moderately effective and ineffective oximes are positioned in the lower part of the plot, characterized by negative PC3 values (Figure 3B). In contrast, highly effective oximes are associated with positive or near-zero PC3 values.

### PC analysis of reactivation for individual organophosphorus compounds

**Figure 5.**
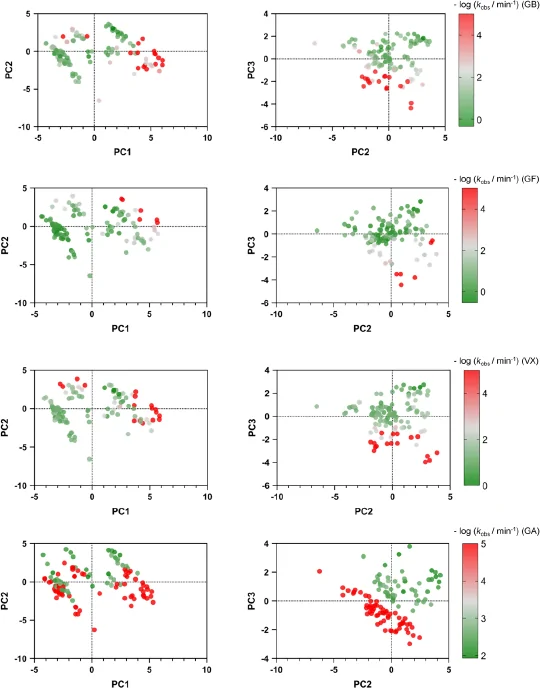
Principal component analysis (PCA) on 115 compounds tested for BChE reactivation after inhibition with sarin (GB), cyclosarin (GF), tabun (GA), and VX. PCA was performed using a dataset comprising 18 variables (Tables S2 to S5). Left row panels represent PC1 *vs*. PC2 correlation, and right row panels represent PC2 *vs.* PC3 correlation. Dark green circles represent the most efficient oximes in the BChE reactivation, and dark red circles represent ineffective oximes in the reactivation.

Individual PC analysis of each OP compound reactivation dataset resulted in 18 PCs, but only three had covariance matrix eigenvectors larger than 1. On average, PC1 contains 49.8 %, PC2 20.0 % and PC3 10.9 % of variance, with matching standard deviations 1.96, 1.0 and 0.53 % (Supplementary material, Tables S2 to S5). This similarity in the corresponding proportions of variance is due to the relatively small number of kinetic variables (four) compared with the 14 pharmacological parameters shared across all four OP compounds. A similar trend is observed in the average values of the eigenvectors for PC1, PC2, and PC3. The values across all four OP compounds were 8.96±0.35, 3.60±0.18 and 1.96±0.09, respectively. The distribution of transformed dataset points is similar among GB, GF and VX data. However, the GA data show a different pattern (Figure 5). PC1 positioned ineffective oxime reactivators on the far right for all four OPs, with the highest positive values (>5). For the GA dataset, ineffective oximes have both PC1 and PC2 negative values. In the correlation of PC2 and PC3, PC3 discriminates oximes by reactivation efficacy. Most of the moderate and ineffective oximes are in the lower part of the graph, having negative PC3 values (Figure 5). Additionally, only PC3 of the GA dataset yielded positive values for ineffective oximes (Figure 5, bottom-right panel).

The most efficient oxime for GB reactivation is **16C**, and it is positioned in the top part of the PC1 *vs.* PC2 correlation plot at coordinates (1.63, 3.07). In contrast, oxime **19D**, which is ineffective, not only for GB reactivation but also for other OPs, is located at coordinates (5.3, 0.05). Additionally, oxime **16C** is the fastest in VX reactivation, and the PC1 *vs.* PC2 correlation positioned it at (1.9, 2.4), while inactive **19D** is found at (5.1, 0.4) coordinates. In the case of GF reactivation, the best reactivator is oxime 4B, and the PC1 *vs.* PC2 correlation positioned it at (1.83, 1.98), while 19D is at coordinates (5.65, 0.84). For GA reactivation, the most efficient oxime **5C** is positioned at (1.46, 1.04), while **19D** is located at (4.4, - 0.1).

**Figure 6.**
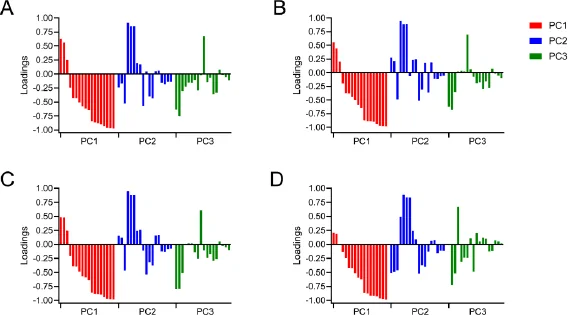
Distribution of loadings from individual PCA using a dataset comprising 18 variables for 115 compounds tested for reactivation of BChE inhibited by sarin (A), cyclosarin (B), VX (C) and tabun (D). Bars are ranked according to the values of PC1 loadings. On average, PC1 contains 46.9 %, PC2 19.1 % and PC3 12.4 % of variance. For further details about the distribution of variables, please see Tables S2 to S5

PC loading analysis reveals consistent patterns after ranking PC1 loadings for each OP compound (Figure 6, Table S2 to S5). The top three loadings are time, h; –log (*k*_obs_ / min^-1^) and molecular fractional polar surface area. These are followed by ADMET Alog *P*98, Alog *P* and log *D* variables, which maintain the same order across all four OP compounds. Negative PC1 loadings also display a consistent order for all four OP compounds: number of H-bond acceptor atoms, ADMET PSA 2D, number of rings, number of aromatic rings, number of rotational bonds, molecular surface area, molecular volume, and molecular weight. Four variable loadings, which are also grouped but whose rank is interchangeable, are dipole magnitude, oxime inhibition of control enzyme activity, %, number of H-bond donor atoms and React_max_, %. The similarity in PC1 loading ranks among OP compounds is attributed to the relatively low number of kinetic variables (four) compared to the fourteen pharmacological parameters shared across all four OP compound analyses.

### ADME results of studied oximes

**Figure 7.**
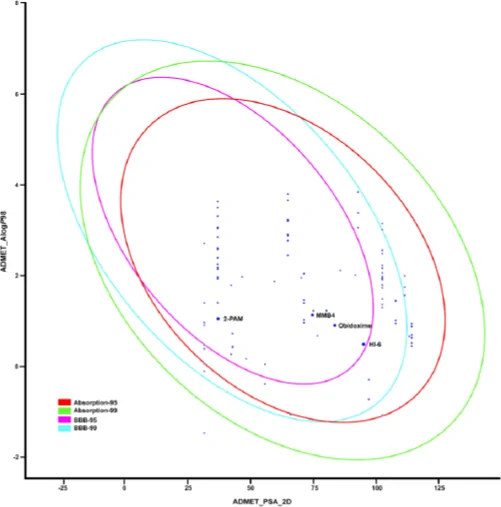
Blood-brain barrier (BBB) permeability plot. ADME (absorption, distribution, metabolism and excretion) properties of the studied compounds were correlated to calculated lipophilicity (Alog *P*98) and polar surface area (PSA 2D) (Table S1). Standard oximes known for efficacy in reactivation of OP-inhibited AChE are 2-PAM, HI-6, obidoxime and TMB-4. The area inside magenta (BBB-95) and red (Absorption-95) ellipses represents compounds with good BBB permeability and human intestinal absorption [24]

The studied oximes were designed and synthesized as potential antidotes for the treatment of OP poisoning and therefore must fulfil the criteria required of viable drug candidates. In addition to sufficient activity toward the therapeutic target, a promising drug candidate must exhibit appropriate absorption, distribution, metabolism, and excretion (ADME) properties. Among these, BBB penetration is one of the most crucial factors, as OP compounds are lipophilic and readily cross biological barriers, including the BBB, thereby inhibiting synaptic AChE [18]. Therefore, the design of effective antidotes should incorporate BBB permeability assessment to identify oximes capable of reactivating ChEs in the CNS. Analyses of numerous approved CNS drugs have led to the establishment of general guidelines describing the physicochemical and ADME properties associated with successful CNS penetration [22]. Favourable physicochemical and ADME properties include adequate oral absorption and sufficient BBB permeability to enable therapeutic concentrations in the brain [23,24]. In this study, the predicted BBB penetration of the oximes was evaluated using a correlation plot of lipophilicity (Alog *P*98) and polar surface area (PSA 2D) (Figure 7). The results show that the majority of the compounds, with the exception of 15 (13 %), fall within the BBB-99 ellipse, indicating increased oxime concentration and residence time in the brain after oral absorption.

A plot of six physicochemical properties of the studied oximes (Supplementary material, Figure S2) was compared to the recommended values for CNS-active drugs. Recommendations include lower molecular weight (MW < 450), moderate hydrophobicity (log *P* < 5), fewer hydrogen bond donors and acceptors (HBD < 3 and HBA < 7), fewer rotatable bonds (RB < 8), and are less polar (polar surface area PSA < 0 .70 nm^2^ (70 Å^2^) [22]. All oximes exhibited optimal values for molecular weight, hydrophobicity, and hydrogen-bond donor and acceptor atom counts. However, 50 compounds (45 %) exceeded the recommended number of rotatable bonds, and 68 compounds (62 %) surpassed the recommended polar surface area threshold (< 0.70 nm^2^). It is generally accepted that a molecule may still exhibit CNS activity if it violates one recommendation criterion. However, it was shown that 47 compounds in the studied library (41 %) do not meet two of the recommendation criteria (Table S1, Figure S2).

## Discussion

In our recent study, a library comprising 115 oximes was tested as reactivators of human BChE inhibited by nerve agents GA, GB, GF, and VX [9]. The determined kinetic parameters were used in the present work, in which PC analysis was applied to integrate them with the physicochemical properties of the oximes and pharmacological parameters relevant to drug design.

PC analysis was applied because of its primary advantage as a non-biased multiple variable method that preserves as much variability as possible while transforming it into new, mutually uncorrelated variables. Application of PC analysis to the reactivation efficacy of selected oximes resulted in three PCs with eigenvector values higher than 1 and a cumulative proportion of variance 72 %. PC1 has a relatively high proportion of variance, 42.4 %. Analysis of PC1 loadings across 27 variables revealed the variables most influential for reactivation efficacy. As expected, the highest positive PC1 loadings were associated with kinetic parameters of BChE reactivation. More interesting, however, was the distribution of loadings for pharmacological parameters (Table 1). Only the molecular fractional polar surface area exhibited positive PC1 loadings, while other parameters showed negative loadings. The ones with values below -0.9 include molecular weight, number of rotational bonds, molecular volume, and molecular surface area. All these parameters can be attributed to the molecule's size.

The correlation between PC1 and PC2 discriminates effective oxime reactivators from moderate or inactive ones. Effective oximes exhibited mainly negative PC1 values and positive PC2 values, while ineffective oximes were characterised by positive PC1 values (>5) and PC2 values (Figure 3). Components PC2 and PC3 jointly account for 29.6 % of the variance, and the PC2 *vs.* PC3 correlation makes the discrimination of oximes by reactivation efficacy more pronounced than the PC1 *vs.* PC2 correlation. Ineffective oximes have negative PC3 values, while effective oximes have positive values or close to zero. PC3 loadings for pharmacological parameters showed that the lowest values were for the number of rotational bonds (0.005), the number of H-bond acceptor atoms (0.088) and the molecular surface area (-0.031). Because the loadings essentially represent the Pearson correlation coefficient, providing a direct measure of their linear relationship, the results of PC3 indicate that three parameters (number of rotational bonds, number of H-bond acceptor atoms, and molecular surface area) have minimal effect on the distribution of transformed data, and consequently, oxime distribution by the reactivation efficacy.

For comparison, separate PCAs were performed for each OP compound. These individual analyses yielded similar proportions of variance for PC1, PC2, and PC3, as well as comparable transformed data distributions or loadings (Tables S2 to S5). Similarity is driven by a higher proportion of the same 14 pharmacological parameters than of the four OP kinetic parameters. The transformed data distribution showed that ineffective oximes were positioned far-right in the PC1 *vs.* PC2 correlation, and effective oximes in the far-left position, except for the GA reactivation dataset. GA reactivation is characterised by a high number of ineffective oximes, which are positioned in both the far-right and far-left. PC2 *vs.* PC3 correlation again more clearly discriminates between effective and ineffective oximes, with effective oximes exhibiting positive PC2 and PC3 values. For GA reactivation, ineffective oximes exhibited both positive and negative PC2 values, with predominantly negative PC3 values. Analysis of PC2 and PC3 loadings for the pharmacological parameters of an individual OP compound indicated that parameters below the 10 % threshold had a minor impact on the distribution of the transformed data. The corresponding variables were the number of aromatic rings and the number of rings for PC2 loadings of GB and GA data, while molecular weight and molecular volume were corresponding variables for GF and VX data.

For PC3 loadings, a larger number of variables (5 to 7) were below the 10 % threshold compared with PC2 loadings. All four OP compounds shared a low, variable molecular volume, while GB, GF and VX data shared the variables: number of rotational bonds and molecular surface area. Molecular volume was present in both PC2 and PC3 components as a low-value variable. When these findings were compared with the joint PC analysis for all four OP compounds, only one PC2 variable (dipole moment) fell below the 10 % threshold. In contrast, several PC3 variables, number of rotational bonds, molecular surface area, and molecular volume, were below this threshold. Interestingly, all low-threshold variables in individual PC analyses are repeated or preserved in the joint PC analysis, indicating overlap between the joint and individual analyses.

A previous study analysing a range of AChE inhibitors (68 compounds) aimed at correlating structural features of inhibitors with overall AChE inhibition potency, showed that inhibition potency was not highly correlated with topological polar surface area (TPSA) or the number of rotational bonds [25]. Yet, the structural features responsible for potent inhibition were molecular weight and the number of atoms. Structure-activity analysis provided insight into how the TPSA parameter, related to PSA 2D, can be linked to the aromatic nature of the AChE active-site gorge, where polar interactions are not required for high ligand affinity. Generally, potent inhibitors are expected to be larger ligands that induce a minor conformational change in the AChE active site residues by forming multiple hydrophobic interactions with aromatic residues of the AChE active site [25].

Variables that represent important pharmacological parameters significant for evaluating a compound’s BBB permeability, and consequently CNS activity, are Alog *P*98 and PSA 2D. According to the BBB plot, 87 % of the oximes in our library were predicted to penetrate the BBB and thus potentially reactivate ChEs in the CNS, accompanied by good human intestinal absorption [24] (*cf.* Figure 7). Loadings calculated from joint PC analysis revealed Alog *P*98 negative values with increasing magnitude: PC1-0.189, PC2-0.333 and PC3-0.870. In contrast, PSA 2D loadings changed from negative values to positive with a decline in the magnitude of PC1-0.814, PC2 0.491, and PC3 0.151. Since PC3 discriminated between ineffective and effective oximes in BChE reactivation, Alog *P*98 loading is important for transforming data distribution and plays a key role in oxime classification. The 2D PSA loading from PC1 influences the distribution of the transformed data. However, PC1 does not discriminate between oximes by reactivation efficacy as effectively as PC3 does.

## Conclusions

To our knowledge, this is the first application of PC analysis to evaluate a library of oximes for efficacy in reactivation of ChE inhibited by OP compounds. Our study confirms the benefit of the PCA method, as it preserves as much variability as possible and translates it into finding new mutually uncorrelated variables that are linear functions of those in the original reactivation dataset. In addition to evaluating oxime efficacy for individual OP compounds (GA, GB, GF and VX), the analysis was expanded to include pharmacokinetic parameters relevant to drug design and BBB permeability, enabling assessment of potential CNS activity. Moreover, structure-activity relationship analysis in the development of novel active compounds with a new scaffold and advanced ADME properties is a widely accepted method [26].

The resulting variable loadings and their magnitudes highlighted significant pharmacokinetic parameters for evaluating oxime efficacy across all four OP compounds. The benefit of simultaneous four OP compound PC analysis over individual OP compound PC analysis lies in the mutual comparison of OP-related kinetic parameters with the addition of pharmacokinetic parameters. A limitation of the individual OP compound PC analysis is the relatively low proportion of kinetic parameters relative to the number of pharmacokinetic parameters included in the analysis, resulting in a very similar distribution of transformed data among OP compounds.

The primary advantage of the joint four OP compounds PC analysis is the ability to identify a single oxime compound, the so-called universal reactivator, that effectively reactivates ChEs inhibited by multiple OP compounds. This is evident in GA reactivation, where many oximes from the studied library were ineffective reactivators. Nevertheless, those that were effective also showed strong reactivation potential of GB-, GF- and VX- BChE conjugate.

BChE also acts as a natural scavenger of OP compounds. Therefore, its reactivation is important for the development of a pseudo-catalytic system in which cycles of BChE inhibition and reactivation with an effective, versatile oxime would degrade OP compounds in a patient's bloodstream prior to inhibition of synaptic AChE. Besides, it would be advantageous if this oxime crossed the BBB and acted as an effective reactivator of BChE in the CNS, thereby protecting synaptic AChE.

## Supplementary material

Additional data are available at https://pub.iapchem.org/ojs/index.php/admet/article/view/3361, or from the corresponding author on request. Plot of three-dimensional data projected into the new coordinate system of principal components is available at https://pub.iapchem.org/ojs/SINKO_PCA_PLOT-3D_scores.html.


