# Supplementary material for: Assessing oxime reactivation efficacy using principal component analysis: Insights from nerve agents inhibited human butyrylcholinesterase
Source: ADMET DMPK. 2026 May 28;14:3361. doi: 10.5599/admet.3361 (PMC13499654; doi:10.5599/admet.3361)
Supplement: Supplementary file 1 [file ADMET-14-3361-S1.pdf]

ADMET & DMPK **14** (2026) S3361

Open Access : ISSN : 1848-7718
http://www.pub.iapchem.org/ojs/index.php/admet

Supplementary material to

Assessing oxime reactivation efficacy using principal component analysis: Insights from nerve agents inhibited human butyrylcholinesterase

Goran Šinko[1], Tena Čadež[1], Zrinka Kovarik[1,2] and Nikolina Maček Hrvat[1]

ADMET & DMPK **14** (2026) 3361; https://doi.org/10.5599/admet.3361

Figure S1. Plot of three-dimensional data projected into the new coordinate system of principal components is available at https://pub.iapchem.org/ojs/SINKO_PCA_PLOT-3D_scores.html

Table S1. Kinetic data for reactivation of BChE inhibited by organophosphorus compounds sarin (GB), cyclorasin (GF), VX and tabun (GA) together with ADME data for a library of 115 oximes including including click-chemistry-derived ones

NAME	SMILES	Oxime inhibition, %	- log (k_{obs} / min^{-1}) (GB)	React$_{max}$, % GB	Time, h (GB)	- log (k_{obs} /min^{-1}) (GF)	React$_{max}$, % (GF)	Time, h (GF)	- log (k_{obs} /min^{-1}) (VX)	React$_{max}$, % (VX)	Time, h (VX)	- log (k_{obs} / min^{-1}) (GA)	React$_{max}$, % (GA)	Time, h (GA)	A log*P*	log*D*	Molecular weight	Number of aromatic rings	Number of H bond acceptor atoms	Number of H bond donor atoms	Number of rings	Number of rotational bonds
1A	[H]C=1N(N=NC=1C([H])([H])C([H])([H])[N+]=2C([H])=C([H])N(C=2C([H])=NO[H])C([H])([H])[H])C([H])([H])C([H])([H])C([H])([H])[N+](C([H])([H])C([H])=NO[H])(C([H])([H])[H])C([H])([H])[H]	46	1.609	86	4.5	0.529	93	0.5	1.393	57	2	5.000	20	24	-0.62	-0.7	364	2	6	2	2	10
2A	[H]/C1=C(/[N+](=C(/[H])C([H])=C1[H])C([H])([H])C([H])([H])C([H])([H])C([H])([H])C=2N=NN(C=2[H])C([H])([H])C3=C([H])C([H])=C([H])C([H])=C3[H])C([H])=NO[H]	36	2.131	81	10	1.381	80	2.25	1.959	61	6.3	2.292	66	10	4.142	4.102	336	3	4	1	3	8
3A	[H]C=1C([H])=C([H])[N+](=C(C=1[H])C([H])=NO[H])C([H])([H])C([H])([H])C=2N=NN(C=2[H])C([H])([H])C([H])([H])C([H])([H])[N+]3=C([H])C([H])=C([H])C([H])=C3C([H])=NO[H]	52	1.061	81	1.75	0.201	70	0.17	1.218	60	1.3	2.033	27	6	3.215	3.158	381	3	6	2	3	9
4A	[H]/C1=[N+](/C([H])=C([H])C(=C1[H])C([H])=NO[H])C([H])([H])C([H])([H])N2N=N/C(=C2/[H])C([H])([H])C([H])([H])[N+]3=C(N(C([H])=C3[H])C([H])([H])[H])C([H])=NO[H]	22	0.745	91	0.5	0.285	73	0.28	1.146	63	1	2.370	32	24	2.039	2.02	370	3	6	2	3	8
5A	[H]C=1N(N=NC=1C([H])([H])C([H])([H])[N+]2=C([H])C([H])=C([H])C([H])=C2C([H])=NO[H])C([H])([H])C([H])([H])C([H])([H])[N+](C([H])([H])C([H])=NO[H])(C([H])([H])[H])C([H])([H])[H]	25	0.945	71	1	0.407	77	0.5	1.320	65	2	5.000	20	24	0.199	0.141	361	2	6	2	2	10
6A	[H]C=1N(N=NC=1C([H])([H])C([H])([H])[N+]2=C([H])C([H])=C([H])C([H])=C2C([H])=NO[H])C([H])([H])OC([H])([H])[N+]3=C(C([H])=C([H])C([H])=C3[H])C([H])=NO[H]	56	0.027	69	0.065	1.340	16	1	0.971	46	3.5	5.000	20	24	2.987	2.919	383	3	7	2	3	9
7A	[H]C=1N(N=NC=1C([H])([H])C([H])([H])[N+](C([H])([H])[H])(C([H])([H])C([H])=NO[H])C([H])([H])[H])C([H])([H])C([H])([H])C([H])([H])[N+]2=C(C([H])=C([H])C([H])=C2[H])C([H])=NO[H]	22	0.884	94	1	0.236	67	0.17	1.340	54	1.3	5.000	20	24	0.199	0.158	361	2	6	2	2	10
8A	[H]C=1N(N=NC=1C([H])([H])C([H])([H])[N+](C([H])([H])[H])(C([H])([H])C([H])=NO[H])C([H])([H])[H])C([H])([H])C([H])([H])C([H])([H])[N+]2=C(N(C([H])=C2[H])C([H])([H])[H])C([H])=NO[H]	24	1.513	72	2.5	1.020	74	3.5	1.561	49	2	5.000	20	24	-0.62	-0.69	364	2	6	2	2	10
9A	[H]C=1N(N=NC=1C([H])([H])C([H])([H])[N+]=2C([H])=C([H])N(C=2C([H])=NO[H])C([H])([H])[H])C([H])([H])C([H])([H])C([H])([H])[N+]3=C(C([H])=C([H])C([H])=C3[H])C([H])=NO[H]	49	1.642	82	4	0.346	64	0.25	1.384	55	1.5	5.000	20	24	2.393	2.32	384	3	6	2	3	9
10A	[H]/C1=C(\[H])[N+](=C(C([H])=C1[H])C([H])=NO[H])C([H])([H])C([H])([H])N2N=N/C(=C2/[H])C([H])([H])C([H])([H])[N+]3=C([H])C([H])=C([H])C([H])=C3C([H])=NO[H]	39	1.027	84	1.75	0.263	83	0.25	1.214	58	1	2.407	24	24	3.153	3.135	367	3	6	2	3	8
11A	[H]/C1=C(\[H])[N+](=C(C([H])=C1[H])C([H])=NO[H])C([H])([H])C([H])([H])C([H])([H])C([H])([H])N2N=N/C(=C2/[H])C([H])([H])[N+]3=C([H])C([H])=C([H])C([H])=C3C([H])=NO[H]	72	1.375	81	2	0.754	66	0.75	1.593	67	14	5.000	20	24	3.547	3.458	381	3	6	2	3	9
12A	[H]C=1N(N=NC=1C([H])([H])C([H])([H])[N+]2=C([H])C([H])=C([H])C([H])=C2C([H])=NO[H])C([H])([H])C([H])([H])C([H])([H])[N+]3=C([H])C([H])=C([H])C([H])=C3[H]	39	1.134	83	1.5	0.498	58	0.17	1.611	66	5.5	5.000	20	24	3.097	3.04	338	3	4	1	3	8

NAME	SMILES	Oxime inhibition, %	- log (k_{obs} / min^{-1}) (GB)	React$_{max}$, % GB	Time, h (GB)	- log (k_{obs} /min^{-1}) (GF)	React$_{max}$, % (GF)	Time, h (GF)	- log (k_{obs} /min^{-1}) (VX)	React$_{max}$, % (VX)	Time, h (VX)	- log (k_{obs} / min^{-1}) (GA)	React$_{max}$, % (GA)	Time, h (GA)	A log*P*	log*D*	Molecular weight	Number of aromatic rings	Number of H bond acceptor atoms	Number of H bond donor atoms	Number of rings	Number of rotational bonds
13A	[H]C=1N(N=NC=1C([H])([H])C([H])([H])[N+]2=C([H])C([H])=C([H])C([H])=C2C([H])=NO[H])C([H])([H])C([H])([H])C([H])([H])[N+]3=C([H])C([H])=C(C([H])=C3[H])C([H])=NO[H]	30	0.628	86	0.5	-0.095	85	0.08	0.951	67	0.8	2.652	54	24	2.924	2.867	381	3	6	2	3	9
14A	[H]/C1=C(/N=NN1C([H])([H])C([H])([H])[N+]=2C([H])=C([H])C(=C([H])C=2[H])C([H])=NO[H])C([H])([H])[N+]3=C([H])C([H])=C([H])C([H])=C3C([H])=NO[H]	31	0.825	87	0.875	0.145	52	0.08	1.034	46	0.8	2.521	27	24	2.613	2.597	353	3	6	2	3	7
15A	O=C(C=1C([H])=C([H])[N+](=C([H])C=1[H])C([H])([H])C([H])([H])N2N=N/C(=C2/[H])C([H])([H])C([H])([H])[N+]3=C([H])C([H])=C([H])C([H])=C3C([H])=NO[H])N([H])[H]	19	1.134	49	0.25	0.400	68	0.5	1.417	93	2	2.706	27	24	2.037	2.019	367	3	5	2	3	8
16A	[H]C=1N(N=NC=1C([H])([H])[N+]2=C([H])C([H])=C([H])C([H])=C2C([H])=NO[H])C([H])([H])C([H])([H])C([H])([H])[N+]3=C(C([H])=C([H])C([H])=C3[H])C([H])=NO[H]	79	1.137	77	2	0.332	81	0.33	1.516	68	2.5	5.000	20	24	2.967	2.949	367	3	6	2	3	8
17A	[H]C=1N(N=NC=1C([H])([H])[N+]2=C([H])C([H])=C([H])C([H])=C2C([H])=NO[H])C([H])([H])OC([H])([H])[N+]3=C(C([H])=C([H])C([H])=C3[H])C([H])=NO[H]	62	0.841	71	1	0.374	58	0.33	1.295	55	2	5.000	20	24	2.738	2.708	369	3	7	2	3	8
18A	[H]/C1=C(/[N+](=C(/[H])C([H])=C1[H])C([H])([H])C([H])([H])C=2N=NN(C=2[H])C([H])([H])C3=C([H])C([H])=C([H])C([H])=C3[H])C([H])=NO[H]	51	2.301	63	17	0.413	65	1.75	2.658	55	24	5.000	20	24	3.365	3.347	308	3	4	1	3	6
19A	[H]/C1=C(\[H])[N+](=C([H])C([H])=C1C([H])=NO[H])C([H])([H])C([H])([H])N2N=N/C(=C2/[H])C([H])([H])C([H])([H])[N+]3=C([H])C([H])=C([H])C([H])=C3C([H])=NO[H]	25	0.757	84	0.75	-0.167	52	0.08	1.187	83	2	2.545	35	24	2.861	2.844	367	3	6	2	3	8
20A	O=C(C=1C([H])=C([H])[N+](=C([H])C=1[H])C([H])([H])C([H])([H])C([H])([H])N2N=N/C(=C2/[H])C([H])([H])C([H])([H])[N+]3=C([H])C([H])=C([H])C([H])=C3C([H])=NO[H])N([H])[H]	29	1.047	79	1	0.465	75	0.5	1.439	67	2	5.000	20	24	2.099	2.042	381	3	5	2	3	9
21A	O=C(C=1C([H])=C([H])[N+](=C([H])C=1[H])C([H])([H])C([H])([H])N2N=N/C(=C2/[H])C([H])([H])[N+]3=C([H])C([H])=C([H])C([H])=C3C([H])=NO[H])N([H])[H]	28	1.148	85	1	0.730	76	0.5	1.650	67	2.5	5.000	20	24	1.789	1.773	353	3	5	2	3	7
22A	[H]/C1=C(\[H])[N+](=C(C([H])=C1[H])C([H])=NO[H])C([H])([H])C([H])([H])N2N=N/C(=C2/[H])C([H])([H])C([H])([H])C([H])([H])[N+]3=C([H])C([H])=C([H])C([H])=C3C([H])=NO[H]	59	1.627	87	3.5	0.616	81	0.75	1.666	60	3.5	2.527	29	24	3.474	3.422	381	3	6	2	3	9
23A	[H]C=1N(N=NC=1C([H])([H])C([H])([H])[N+]2=C([H])C([H])=C([H])C([H])=C2C([H])=NO[H])C([H])([H])C([H])([H])C([H])([H])[N+]3=C(N(C([H])=C3[H])C([H])([H])[H])C([H])=NO[H]	52	1.633	86	5	0.112	57	0.17	1.688	52	2.3	2.435	40	24	2.393	2.322	384	3	6	2	3	9
24A	O=C(/C1=C(\[H])C([H])=[N+](C([H])=C1[H])C([H])([H])C([H])([H])C([H])([H])N2N=N/C(=C2/[H])C([H])([H])[N+]=3C([H])=C([H])C([H])=C([H])C=3C([H])=NO[H])N([H])[H]	53	1.613	80	4	0.696	58	0.5	2.027	57	3.5	5.000	20	24	1.851	1.835	367	3	5	2	3	8
25A	[H]C=1N(N=NC=1C([H])([H])C([H])([H])[N+]2=C([H])C([H])=C(C([H])=C2[H])C([H])=NO[H])C([H])([H])OC([H])([H])[N+]3=C([H])C([H])=C([H])C([H])=C3C([H])=NO[H]	42	1.309	64	2	0.342	70	0.33	1.907	57	7.5	5.000	20	24	2.695	2.627	383	3	7	2	3	9
26A	[H]C=1N(N=NC=1C([H])([H])C([H])([H])[N+]2=C([H])C([H])=C([H])C([H])=C2[H])C([H])([H])C([H])([H])C([H])([H])[N+]3=C([H])C([H])=C([H])C([H])=C3C([H])=NO[H]	49	1.123	87	1.5	0.794	62	0.5	1.439	82	3	5.000	20	24	3.097	3.056	338	3	4	1	3	8
27A	O=C(C=1C([H])=C([H])[N+](=C([H])C=1[H])C([H])([H])C([H])([H])C([H])([H])N2N=N/C(=C2/[H])C([H])([H])C([H])([H])[N+]3=C([H])C([H])=C(C([H])=C3[H])C([H])=NO[H])N([H])[H]	27	0.963	82	1	-0.211	55	0.08	1.429	60	2	2.636	41	24	1.808	1.805	381	3	5	2	3	9
28A	[H]/C1=C(\[H])[N+](=C(C([H])=C1[H])C([H])=NO[H])C([H])([H])C([H])([H])N2N=N/C(=C2/[H])C([H])([H])C([H])([H])[N+]3=C([H])C([H])=C(C([H])=C3[H])C([H])=NO[H]	24	0.915	92	1	-0.045	80	0.08	1.192	54	1.5	2.400	20	24	2.861	2.845	367	3	6	2	3	8
29A	[H]/C1=C(/[N+](=C(/[H])C([H])=C1[H])C([H])([H])C([H])([H])C([H])([H])C=2N=NN(C=2[H])C([H])([H])C3=C([H])C([H])=C([H])C([H])=C3[H])C([H])=NO[H]	35	2.301	72	24	1.405	73	2.25	2.585	52	24	2.251	66	10	3.686	3.668	322	3	4	1	3	7
30A	O=C(/C1=C(\[H])C([H])=[N+](C([H])=C1[H])C([H])([H])C([H])([H])C=2N=NN(C=2[H])C([H])([H])C([H])([H])[N+]=3C([H])=C([H])C([H])=C([H])C=3C([H])=NO[H])N([H])[H]	24	1.130	85	1.25	0.598	65	0.5	1.635	76	2.3	2.614	22	24	2.037	2.021	367	3	5	2	3	8
31A	[H]C=1N(N=NC=1C([H])([H])C([H])([H])[N+]=2C([H])=C([H])N(C=2C([H])=NO[H])C([H])([H])[H])C([H])([H])C([H])([H])C([H])([H])[N+]3=C([H])C([H])=C(C([H])=C3[H])C([H])=NO[H]	46	0.866	83	1.5	0.075	70	0.17	0.964	64	1.5	2.793	48	24	2.101	2.029	384	3	6	2	3	9
32A	[H]C([H])([N+]=1C([H])=C([H])C([H])=C([H])C=1C([H])=NO[H])C([H])([H])N2N=N/C(=C2/[H])C([H])([H])C([H])([H])C([H])([H])[N+]3=C([H])C([H])=C(C([H])=C3[H])C([H])=NO[H]	39	0.781	86	0.625	0.323	92	0.33	1.166	67	2	2.793	31	24	3.183	3.132	381	3	6	2	3	9
33A	O=C(/C1=C(\[H])C([H])=[N+](C([H])=C1[H])C([H])([H])C([H])([H])C=2N=NN(C=2[H])C([H])([H])C([H])([H])[N+]=3C([H])=C([H])C(=C([H])C=3[H])C([H])=NO[H])N([H])[H]	17	1.194	79	1.25	0.157	53	0.17	1.264	54	1.3	2.523	30	24	1.746	1.743	367	3	5	2	3	8
34A	[H]/C1=[N+](/C(=C(/[H])C([H])=C1[H])C([H])=NO[H])C([H])([H])C([H])([H])C=2N=NN(C=2[H])C([H])([H])N3N=N/C(=C3/[H])C4=C([H])C([H])=C([H])C([H])=C4[H]	17	2.921	58	14.5	2.252	63	17	2.569	33	24	5.000	20	24	3.542	3.524	375	4	6	1	4	7
35A	O=C(/C1=C(\[H])C([H])=[N+](C([H])=C1[H])C([H])([H])C([H])([H])C=2N=NN(C=2[H])C([H])([H])C([H])([H])C([H])([H])[N+]3=C([H])C([H])=C([H])C([H])=C3C([H])=NO[H])N([H])[H]	27	1.241	86	1.75	0.588	60	0.5	1.747	75	2.5	5.000	20	24	2.099	2.059	381	3	5	2	3	9
36A	O=C(C=1C([H])=C([H])[N+](=C([H])C=1[H])C([H])([H])C([H])([H])N2N=N/C(=C2/[H])C([H])([H])C([H])([H])[N+]3=C([H])C([H])=C(C([H])=C3[H])C([H])=NO[H])N([H])[H]	19	1.044	87	1	0.245	55	0.17	1.268	60	1.5	2.721	44	24	1.746	1.743	367	3	5	2	3	8

CC BY

NAME	SMILES	Oxime inhibition, %	- log (k_{obs} / min^{-1}) (GB)	React$_{max}$, % GB	Time, h (GB)	- log (k_{obs} /min^{-1}) (GF)	React$_{max}$, % (GF)	Time, h (GF)	- log (k_{obs} /min^{-1}) (VX)	React$_{max}$, % (VX)	Time, h (VX)	- log (k_{obs} / min^{-1}) (GA)	React$_{max}$, % (GA)	Time, h (GA)	A log*P*	log*D*	Molecular weight	Number of aromatic rings	Number of H bond acceptor atoms	Number of H bond donor atoms	Number of rings	Number of rotational bonds
37A	[H]/C1=C(\[H])[N+](=C([H])C([H])=C1C([H])=NO[H])C([H])([H])C([H])([H])N2N=N/C(=C2/[H])C([H])([H])C([H])([H])[N+]3=C([H])C([H])=C(C([H])=C3[H])C([H])=NO[H]	15	0.573	84	0.5	0.057	57	0.17	1.093	76	1.3	2.717	53	24	2.57	2.567	367	3	6	2	3	8
38A	[H]/C1=C(\[H])[N+](=C(C([H])=C1[H])C([H])=NO[H])C([H])([H])C([H])([H])C([H])([H])C([H])([H])C=2N=NN(C=2[H])C([H])([H])C([H])([H])C([H])([H])[N+]3=C(C([H])=C([H])C([H])=C3[H])C([H])=NO[H]	68	1.287	88	2.25	0.234	75	0.17	1.775	57	3.5	5.000	20	24	3.993	3.952	409	3	6	2	3	11
39A	[H]/C1=[N+](/C(=C(/[H])C([H])=C1[H])C([H])=NO[H])C([H])([H])C([H])([H])C([H])([H])C([H])([H])C=2N=NN(C=2[H])C([H])([H])N3N=N/C(=C3/[H])C4=C([H])C([H])=C([H])C([H])=C4[H]	26	5.000	20	14.5	2.347	64	24	5.000	20	24	2.317	71	19.3	4.319	4.279	403	4	6	1	4	9
40A	[H]C=1N(N=NC=1C([H])([H])C([H])([H])[N+]2=C([H])C([H])=C(C([H])=C2[H])C([H])=NO[H])C([H])([H])C([H])([H])C([H])([H])[N+]3=C([H])C([H])=C([H])C([H])=C3C([H])=NO[H]	28	0.860	93	0.875	0.135	70	0.17	1.350	65	2.2	5.000	20	24	2.924	2.883	381	3	6	2	3	9
41A	[F]C=1C([H])=[N+](C([H])=C([H])C=1C([H])=NO[H])C([H])([H])C([H])([H])N2N=N/C(=C2/[H])C([H])([H])C([H])([H])[N+]3=C([H])C([H])=C([H])C([H])=C3C([H])=NO[H]	27	1.349	89	1.75	0.504	64	0.5	1.611	65	2.5	2.697	28	24	3.067	3.049	385	3	6	2	3	8
42A	[H]C=1N(N=NC=1C([H])([H])C([H])([H])[N+](C([H])([H])[H])(C([H])([H])C([H])=NO[H])C([H])([H])[H])C([H])([H])C([H])([H])C([H])([H])[N+]2=C([H])C([H])=C(C([H])=C2[H])C([H])=NO[H]	21	0.853	92	1	0.915	66	1	1.085	67	1.5	2.550	53	24	-0.09	-0.1	361	2	6	2	2	10
43A	[F]C=1/C(=C(/[H])C([H])=[N+](C=1[H])C([H])([H])C([H])([H])C=2N=NN(C=2[H])C([H])([H])C([H])([H])[N+]=3C([H])=C([H])C([H])=C([H])C=3C([H])=NO[H])C([H])=NO[H]	25	0.940	84	0.875	0.298	74	0.17	1.330	62	1.8	5.000	20	24	3.067	3.051	385	3	6	2	3	8
44A	[H]C=1N(N=NC=1C([H])([H])C([H])([H])[N+]=2C([H])=C([H])N(C=2C([H])=NO[H])C([H])([H])[H])C([H])([H])C([H])([H])C([H])([H])[N+]3=C(N(C([H])=C3[H])C([H])([H])[H])C([H])=NO[H]	60	1.903	83	6	0.564	67	0.625	1.896	60	4	5.000	20	24	1.57	1.498	387	3	6	2	3	9
45A	[H]C=1N(N=NC=1C([H])([H])C([H])([H])[N+]2=C([H])C([H])=C(C([H])=C2[H])C([H])=NO[H])C([H])([H])C([H])([H])C([H])([H])[N+]3=C([H])C([H])=C(C([H])=C3[H])C([H])=NO[H]	17	0.306	90	0.17	-0.133	88	0.08	0.959	55	1	2.903	46	24	2.632	2.63	381	3	6	2	3	9
46A	[H]C=1N(N=NC=1C([H])([H])C([H])([H])[N+](C([H])([H])[H])(C([H])([H])C([H])=NO[H])C([H])([H])[H])C([H])([H])C([H])([H])[N+](C([H])([H])C([H])=NO[H])(C([H])([H])[H])C([H])([H])[H]	19	2.585	89	24	0.886	64	1	1.618	55	3	5.000	20	24	-2.82	-2.82	341	1	6	2	1	11
47A	[H]/C1=C(\[H])[N+](=C([H])C([H])=C1C([H])=NO[H])C([H])([H])C([H])([H])C([H])([H])C=2N=NN(C=2[H])C([H])([H])C3=C([H])C([H])=C([H])C([H])=C3[H]	31	1.326	55	2	1.135	55	1.875	1.530	48	3	2.609	20	24	3.395	3.392	322	3	4	1	3	7
48A	[H]C=1N(N=NC=1C([H])([H])C([H])([H])[N+]2=C([H])C([H])=C(C([H])=C2[H])C([H])=NO[H])C([H])([H])C([H])([H])C([H])([H])[N+](C([H])([H])C([H])=NO[H])(C([H])([H])[H])C([H])([H])[H]	19	1.093	75	1	0.214	79	0.25	1.265	63	3.3	5.000	20	24	-0.09	-0.1	361	2	6	2	2	10
49A	[H]/C1=C(\[H])[N+](=C(C([H])=C1[H])C([H])=NO[H])C([H])([H])C([H])([H])C([H])([H])N2N=N/C(=C2/[H])C3=C([H])C([H])=C([H])C([H])=C3[H]	86	5.000	20	14	1.754	45	14.5	5.000	20	24	5.000	20	24	3.393	3.375	308	3	4	1	3	6
50A	[H]C([H])([N+]=1C([H])=C([H])C([H])=C([H])C=1C([H])=NO[H])C([H])([H])N2N=N/C(=C2/[H])C([H])([H])[N+]3=C([H])C([H])=C([H])C([H])=C3C([H])=NO[H]	48	0.877	82	1.5	0.174	77	0.17	1.394	71	2	5.000	20	24	2.905	2.889	353	3	6	2	3	7
51A	[H]/C1=C(\[H])C([H])=C([H])C(=[N+]1C([H])([H])C([H])([H])C#CC([H])([H])C=2N=NN(C=2[H])C([H])([H])C([H])([H])C([H])([H])[N+]3=C([H])C([H])=C([H])C([H])=C3C([H])=NO[H])C([H])=NO[H]	60	1.199	82	1.75	0.178	85	0.21	1.788	60	14.5	5.000	20	24	4.127	4.067	419	3	6	2	3	11
62A	[H]/C1=C(\[H])[N+](=C([H])C([H])=C1C([H])=NO[H])C([H])([H])C([H])([H])C=2N=NN(C=2[H])C([H])([H])C3=C([H])C([H])=C([H])C([H])=C3[H]	25	0.819	69	1.5	1.360	70	2	1.460	53	2.3	5.000	20	24	3.074	3.071	308	3	4	1	3	6
53A	[H]/C1=C(/C([H])=C([H])C([H])=[N+]1C([H])([H])C([H])([H])C=2N=NN(C=2[H])C([H])([H])C3=C([H])C([H])=C([H])C([H])=C3[H])C([H])=NO[H]	27	1.324	74	2	0.422	72	0.5	1.425	60	2.3	5.000	20	24	3.074	3.069	308	3	4	1	3	6
54A	[H]C([H])([N+]=1C([H])=C([H])C(=C([H])C=1[H])C([H])=NO[H])C([H])([H])N2N=N/C(=C2/[H])C([H])([H])C([H])([H])C([H])([H])[N+]3=C([H])C([H])=C([H])C([H])=C3C([H])=NO[H]	33	0.759	81	0.75	0.451	59	0.17	1.293	73	1.8	2.332	21	24	3.183	3.143	381	3	6	2	3	9
55A	[H]/C1=C(/C([H])=C([H])C([H])=[N+]1C([H])([H])C([H])([H])C([H])([H])C=2N=NN(C=2[H])C([H])([H])C3=C([H])C([H])=C([H])C([H])=C3[H])C([H])=NO[H]	42	1.224	70	2	0.536	63	0.625	1.474	56	2.8	2.360	38	2	3.395	3.39	322	3	4	1	3	7
56A	[H]/C1=C(\[H])[N+](=C(C([H])=C1[H])C([H])=NO[H])C([H])([H])C([H])([H])C([H])([H])C=2N=NN(C=2[H])C([H])([H])C([H])([H])C([H])([H])[N+]3=C([H])C([H])=C([H])C([H])=C3C([H])=NO[H]	70	1.699	82	4	0.275	80	0.21	2.114	47	4.5	5.000	20	24	3.536	3.496	395	3	6	2	3	10
57A	O=C(/C1=C(\[H])C([H])=[N+](C([H])=C1[H])C([H])([H])C([H])([H])C=2N=NN(C=2[H])C([H])([H])C([H])([H])C([H])([H])[N+]3=C([H])C([H])=C(C([H])=C3[H])C([H])=NO[H])N([H])[H]	20	0.816	78	0.625	0.184	69	0.17	1.051	66	1.1	2.491	43	24	1.808	1.805	381	3	5	2	3	9
58A	[H]/C1=C(\[H])[N+](=C([H])C([H])=C1C([H])=NO[H])C([H])([H])C([H])([H])C([H])([H])C([H])([H])C=2N=NN(C=2[H])C([H])([H])C3=C([H])C([H])=C([H])C([H])=C3[H]	29	1.900	58	5.5	1.636	54	4.25	1.910	54	6.5	5.000	20	24	3.851	3.848	336	3	4	1	3	8
59A	[H]/C1=C(/C([H])=C([H])C([H])=[N+]1C([H])([H])C([H])([H])C([H])([H])C([H])([H])C=2N=NN(C=2[H])C3=C([H])C([H])=C([H])C([H])=C3[H])C([H])=NO[H]	53	2.086	69	10	0.782	59	0.5	1.870	56	7	5.000	20	24	3.844	3.839	322	3	4	1	3	7

NAME	SMILES	Oxime inhibition, %	- log (k_{obs} / min^{-1}) (GB)	React$_{max}$, % GB	Time, h (GB)	- log (k_{obs} /min^{-1}) (GF)	React$_{max}$, % (GF)	Time, h (GF)	- log (k_{obs} /min^{-1}) (VX)	React$_{max}$, % (VX)	Time, h (VX)	- log (k_{obs} / min^{-1}) (GA)	React$_{max}$, % (GA)	Time, h (GA)	A log$P$	log$D$	Molecular weight	Number of aromatic rings	Number of H bond acceptor atoms	Number of H bond donor atoms	Number of rings	Number of rotational bonds
60A	[H]/C1=[N+](/C(=C(/[H])C([H])=C1[H])C([H])=NO[H])C([H])([H])C([H])([H])C([H])([H])C=2N=NN(C=2[H])C([H])([H])N3N=N/C(=C3/[H])C4=C([H])C([H])=C([H])C([H])=C4[H]	30	2.367	74	9.5	1.719	61	1.75	5.000	20	24	2.498	39	24	3.863	3.845	389	4	6	1	4	8
61A	[H]/C1=C(\[H])[N+](=C(C([H])=C1[H])C([H])=NO[H])C([H])([H])C([H])([H])C#CC([H])([H])C=2N=NN(C=2[H])C([H])([H])C3=C([H])C([H])=C([H])C([H])=C3[H]	56	2.721	50	12	2.409	69	24	5.000	20	24	5.000	20	24	4.277	4.216	346	3	4	1	3	8
62A	[H]C=1N(N=NC=1C([H])([H])C([H])([H])[N+]2=C([H])C([H])=C(C([H])=C2[H])C([H])=NO[H])C([H])([H])C([H])([H])C([H])([H])[N+]3=C(N(C([H])=C3[H])C([H])([H])[H])C([H])=NO[H]	39	1.479	84	3.5	0.286	53	0.17	1.529	55	4.5	5.000	20	24	2.101	2.031	384	3	6	2	3	9
1B	[H]/C1=C(/[N+](=C(/[H])C([H])=C1[H])C([H])([H])C([H])([H])C([H])([H])[H])C([H])=NO[H]	11	0.859	86	1.25	0.379	75	0.585	0.855	66	0.6	2.567	41	24	2.425	2.36	165	1	2	1	1	3
2B	[H]/C1=C(/[N+](=C(/[H])C([H])=C1[H])C([H])([H])C([H])([H])C([H])([H])C([H])([H])[H])C([H])=NO[H]	13	0.320	81	0.21	0.440	79	0.25	1.221	59	0.7	2.620	36	24	2.881	2.821	179	1	2	1	1	4
3B	[H]/C1=C(/[N+](=C(/[H])C([H])=C1[H])C([H])([H])C([H])([H])[H])C([H])=NO[H]	11	1.526	88	3	1.378	66	2.5	1.233	68	1.5	5.000	20	24	1.901	1.842	151	1	2	1	1	2
4B	[H]/C1=C(/[N+](=C(/[H])C([H])=C1[H])C([H])([H])C([H])([H])C([H])([H])C([H])([H])C([H])([H])[H])C([H])=NO[H]	35	0.537	73	0.18	-0.538	60	0.118	1.292	53	1.3	2.292	35	13	3.337	3.277	193	1	2	1	1	5
5B	[H]/C1=C(/[N+](=C(/[H])C([H])=C1[H])C([H])([H])C([H])([H])C([H])([H])C([H])([H])C([H])([H])C([H])([H])[H])C([H])=NO[H]	37	0.801	85	0.5	-0.456	79	0.033	0.454	65	0.2	2.210	44	6.7	3.793	3.733	207	1	2	1	1	6
1C	[N-]=[N+]=NC([H])([H])C([H])([H])C([H])([H])[N+]=1/C(=C(/[H])C([H])=C([H])C=1[H])C([H])=NO[H]	19	0.725	78	0.75	0.284	76	10	1.216	61	2	2.703	40	24	2.377	2.213	206	1	4	1	1	5
2C	[N-]=[N+]=NC([H])([H])C([H])([H])[N+]=1C([H])=C([H])C([H])=C([H])C=1C([H])=NO[H]	29	1.369	82	2	1.163	76	1.5	2.456	48	24	2.714	29	24	2.315	2.171	192	1	4	1	1	4
3C	[H]/C1=C(\[H])[N+](=C([H])C([H])=C1C([H])=NO[H])C([H])([H])C([H])([H])C([H])([H])C#C[H]	14	0.780	63	1	1.654	76	4	1.914	63	8	2.812	28	24	3.23	3.175	189	1	2	1	1	4
4C	[N-]=[N+]=NC([H])([H])OC([H])([H])[N+]=1C([H])C([H])C([H])=C([H])C=1C([H])=NO[H]	39	0.803	76	1	0.969	46	1	2.553	38	24	2.556	27	24	2.148	1.945	208	1	5	1	1	5
5C	[N-]=[N+]=NC([H])([H])C([H])([H])C([H])([H])C([H])([H])[N+]=1C([H])=C([H])C([H])=C([H])C=1C([H])=NO[H]	18	2.658	60	24	1.021	73	1.5	2.408	38	24	1.928	14	17	2.957	2.793	220	1	4	1	1	6
6C	[H]/C1=C(/[N+](=C(/[H])C([H])=C1[H])C([H])([H])C([H])([H])C([H])([H])C#C[H])C([H])=NO[H]	24	0.935	87	1	0.559	67	0.375	1.652	60	4	2.654	43	24	3.522	3.462	189	1	2	1	1	4
7C	[H]/C1=C(/[N+](=C(/[H])C([H])=C1[H])C([H])([H])C([H])([H])C([H])([H])C([H])([H])C#C[H])C([H])=NO[H]	27	1.181	78	2	5.000	35	0.017	1.724	56	4.5	2.352	39	10	3.978	3.918	203	1	2	1	1	5
8C	[H]/C1=C(/[N+](=C(/[H])C([H])=C1[H])C([H])([H])C([H])([H])C#C[H])C([H])=NO[H]	25	1.350	73	2	1.452	73	2.5	2.000	52	8	5.000	20	24	3.066	3.005	175	1	2	1	1	3
9C	[H]/C1=C(/[N+](=C(/[H])C([H])=C1[H])C([H])([H])C([H])([H])C#CC([H])([H])C#C[H])C([H])=NO[H]	21	0.920	83	0.875	5.000	40	0.017	1.742	62	1.8	2.726	37	24	4.112	4.052	213	1	2	1	1	5
10C	[H]/C1=C(\[H])[N+](=C([H])C([H])=C1C([H])=NO[H])C([H])([H])C([H])([H])C([H])([H])C([H])([H])C#C[H]	17	1.023	78	1	0.666	71	0.4	1.642	56	4.5	2.623	36	24	3.687	3.631	203	1	2	1	1	5
11C	[H]/C1=C(/C([H])=C([H])C([H])=[N+]1C([H])([H])C([H])([H])C([H])([H])C#C[H])C([H])=NO[H]	15	0.192	71	0.17	0.784	64	0.5	0.311	40	0.2	2.074	54	10	3.23	3.228	189	1	2	1	1	4
12C	[N-]=[N+]=NC([H])([H])C([H])([H])C([H])([H])[N+]=1C([H])=C([H])C(=C([H])C=1[H])C([H])=NO[H]	19	1.168	88	1.5	0.383	69	0.3	1.572	57	3.5	5.000	20	24	2.086	1.926	206	1	4	1	1	5
13C	[N-]=[N+]=NC([H])([H])C([H])([H])[N+]=1C([H])=C([H])C(=C([H])C=1[H])C([H])=NO[H]	14	1.223	84	1.5	0.103	45	0.2	1.447	59	4.7	2.660	24	24	2.023	1.864	192	1	4	1	1	4
14C	[H]/C1=C(/C([H])=C([H])C([H])=[N+]1C([H])([H])C([H])([H])C#C[H])C([H])=NO[H]	13	1.333	60	2	1.620	63	4	0.507	26	0.6	5.000	20	24	2.774	2.772	175	1	2	1	1	3
15C	[H]/C1=C(\[H])[N+](=C([H])C([H])=C1C([H])=NO[H])C([H])([H])C([H])([H])C#C[H]	11	0.692	54	1	1.724	70	7	2.066	51	4.5	2.635	43	24	2.774	2.718	175	1	2	1	1	3
16C	[H]/C1=C(/C([H])=C([H])C([H])=[N+]1C([H])([H])C([H])([H])C([H])([H])C([H])([H])C#C[H])C([H])=NO[H]	24	-0.335	64	0.17	0.291	66	0.2	-0.002	38	0.1	2.088	60	13.3	3.687	3.684	203	1	2	1	1	5
17C	[H]/C1=C(\[H])[N+](=C(C([H])=C1[H])C([H])=NO[H])C([H])([H])C#C[H]	47	0.531	65	0.5	0.407	71	0.3	5.000	20	24	5.000	20	24	2.744	2.687	161	1	2	1	1	2
1D	[H]/C(=N/O[H])C=1[N+](=NN(C=1C([H])=NO[H])C([H])([H])C2=C([H])C([H])=C([H])C([H])=C2[H])C([H])([H])[H]	24	1.381	84	3	1.133	62	1.75	1.330	82	9	2.567	74	24	2.693	2.672	260	2	5	2	2	4
2D	[H]/C1=C(\[H])C([H])=C2/C(=C1/[H])C(=C([H])C([H])=[N+]2C([H])([H])[H])C([H])=NO[H]	20	1.116	72	2	1.124	55	1.75	1.796	76	4.5	2.699	65	24	2.461	2.42	187	2	2	1	2	1
3D	[H]/C1=C(\[H])[N+](=C(N1C([H])([H])[H])C([H])=NO[H])C([H])([H])C([H])([H])C#C[H]	23	1.243	81	2	0.509	59	0.33	1.147	63	1.5	5.000	20	24	2.243	2.172	178	1	2	1	1	3
4D	[N-]=[N+]=NC([H])([H])C([H])([H])C([H])([H])[N+]=1C([H])=C([H])N(C=1C([H])=NO[H])C([H])([H])[H]	25	0.583	85	0.335	0.355	77	0.33	0.786	62	1	2.851	32	24	1.555	1.354	209	1	4	1	1	5
5D	[N-]=[N+]=NC([H])([H])C([H])([H])C([H])([H])[N+](C([H])([H])C([H])=NO[H])(C([H])([H])[H])C([H])([H])[H]	16	2.319	52	15	0.943	69	1	1.860	46	5	5.000	20	24	-0.64	-0.75	186	0	4	1	0	6
6D	[H]C([H])(C([H])=NO[H])[N+](C([H])([H])[H])(C([H])([H])[H])C([H])([H])[H]	21	3.301	38	24	1.391	71	2	2.194	38	10	5.000	20	24	-1.46	-1.47	117	0	2	1	0	2
7D	O=C(/C1=[N+](/C([H])=C([H])C([H])=C1[H])C([H])([H])[H])N([H])O[H]	19	5.000	20	24	2.260	65	17.5	2.699	35	24	5.000	20	24	0.74	0.508	153	1	2	2	1	1
8D	O=C(C=1C([H])=C([H])[N+](=C([H])C=1[H])C([H])([H])[H])N([H])O[H]	15	5.000	20	24	2.319	50	17.5	5.000	20	24	5.000	20	24	0.449	0.412	153	1	2	2	1	1
9D	O=C(C=1C([H])=[N+](C([H])=C([H])C=1[H])C([H])([H])[H])N([H])O[H]	12	5.000	20	24	1.893	54	6	5.000	20	24	5.000	20	24	0.449	-0.1	153	1	2	2	1	1
10D	[H]/C(=N/O[H])C=1N=NN(C=1C([H])=NO[H])C([H])([H])C2=C([H])C([H])=C([H])C([H])=C2[H]	12	2.678	34	24	2.060	47	6.5	2.432	36	24	5.000	20	24	1.969	1.961	245	2	6	2	2	4
11D	[H]C1([H])C(=NO[H])C([H])([H])[S+](C1([H])[H])C([H])([H])[H]	14	5.000	20	24	2.252	23	17	5.000	20	24	5.000	20	24	0.48	0.477	132	0	2	1	1	0
12D	[H]C=1[S]C(=NC=1C([H])([H])[H])C([H])=NO[H]	14	5.000	20	24	1.767	60	4	2.553	38	24	5.000	20	24	0.801	0.655	142	1	3	1	1	1
13D	[H]C([H])(C([H])=NO[H])[N+](C([H])([H])[H])(C([H])([H])C([H])([H])C#C[H])C([H])([H])[H]	18	2.569	68	24	0.888	70	1	1.684	30	2.1	5.000	20	24	0.049	0.045	155	0	2	1	0	4

(cc) BY

NAME	SMILES	Oxime inhibition, %	- log (k_{obs} / min^{-1}) (GB)	React$_{max}$, % GB	Time, h (GB)	- log (k_{obs} /min^{-1}) (GF)	React$_{max}$, % (GF)	Time, h (GF)	- log (k_{obs} /min^{-1}) (VX)	React$_{max}$, % (VX)	Time, h (VX)	- log (k_{obs} / min^{-1}) (GA)	React$_{max}$, % (GA)	Time, h (GA)	A log*P*	log*D*	Molecular weight	Number of aromatic rings	Number of H bond acceptor atoms	Number of H bond donor atoms	Number of rings	Number of rotational bonds
14D	[H]/C1=C(/C([H])=C([H])[S+]1C([H])([H])[H])C([H])=NO[H]	13	5.000	20	24	5.000	20	24	5.000	20	24	5.000	20	24	1.42	1.417	142	1	2	1	1	1
15D	[H]C=1N=C([S]C=1[H])C(=NO[H])C([H])([H])[H]	15	2.538	40	24	0.887	27	1	5.000	20	24	5.000	20	24	0.499	0.498	142	1	3	1	1	1
16D	[H]C=1[N+](=C(N(C=1[H])C([H])([H])[H])C([H])=NO[H])C([H])([H])[H]	15	1.308	83	3	1.177	66	1.5	1.618	65	4	5.000	20	24	0.73	0.657	140	1	2	1	1	1
17D	[F]C=1C([F])=C([F])C([F])=C(C=1[F])C([H])=NO[H]	23	5.000	20	24	2.276	70	24	2.481	22	5	5.000	20	24	2.684	2.683	211	1	2	1	1	1
18D	[H]/C1=C(/N=C(C([H])=C1[H])C([H])=NO[H])C([H])=NO[H]	16	5.000	20	24	1.866	39	5	5.000	20	24	5.000	20	24	1.189	1.234	165	1	5	2	1	2
19D	[H]/C1=C(/[S+](C([H])=C1[H])C([H])([H])[H])C([H])=NO[H]	14	5.000	20	24	5.000	20	24	5.000	20	24	5.000	20	24	1.712	1.712	142	1	2	1	1	1
20D	[H]C1([H])[S]C([H])([H])C([H])([H])C1=NO[H]	32	5.000	20	24	2.060	63	10	5.000	20	24	5.000	20	24	0.378	0.378	117	0	3	1	1	0
21D	[H]C=1N=C(N(C=1[H])C([H])([H])[H])C([H])=NO[H]	22	2.367	71	24	1.090	43	0.625	1.684	38	3.5	5.000	20	24	0.156	0.266	125	1	3	1	1	1
22D	[H]/C1=C(\[H])C([H])=C2/C(=C1/[H])C(=C([H])N2[H])C([H])=NO[H]	22	5.000	20	24	5.000	20	24	5.000	20	24	5.000	20	24	1.95	1.949	160	2	2	2	2	1
23D	[H]C=1N=C([S]C=1[H])C([H])=NO[H]	16	2.569	55	24	1.133	59	1.75	2.387	42	24	5.000	20	24	0.518	0.517	128	1	3	1	1	1
24D	[H]C=1[S]C([H])=C([H])C=1C([H])=NO[H]	14	5.000	20	22	2.602	25	17.5	5.000	20	24	5.000	20	24	1.318	1.317	127	1	2	1	1	1
25D	[H]/C1=C(/[S]C([H])=C1[H])C([H])=NO[H]	8	5.000	20	12	2.553	43	24	5.000	20	24	5.000	20	24	1.61	1.609	127	1	2	1	1	1
26D	[H]/C1=C(\[H])C([H])=C2/C(=C1/[H])C(=C([H])N2C([H])([H])[H])C([H])=NO[H]	25	5.000	20	24	5.000	20	24	5.000	20	24	5.000	20	24	2.156	2.154	174	2	2	1	2	1
27D	[H]C=1N(N=NC=1C([H])=NO[H])C([H])([H])C2=C([H])C([H])=C([H])C([H])=C2[H]	16	5.000	20	24	2.125	74	11	2.824	48	24	5.000	20	24	1.851	1.847	202	2	4	1	2	3
2-PAM	[H]C=1C([H])=[N+](/C(=C(/[H])C=1[H])C([H])=NO[H])C([H])([H])[H]	8	0.872	80	1.357	1.054	72	1	1.249	56	1.7	2.670	27	12	1.552	1.45	137	1	2	1	1	1
HI-6	O=C(/C1=C(\[H])C([H])=[N+](C([H])=C1[H])C([H])([H])OC([H])([H])[N+]2=C([H])C([H])=C([H])C([H])=C2C([H])=NO[H])N([H])[H]	11	0.959	92	0.6667	-0.050	71	0.17	1.418	62	1.8	2.194	14	5	1.641	1.373	288	2	4	2	2	6
Obid-oxime	[H]/C1=[N+](/C([H])=C([H])C(=C1[H])C([H])=NO[H])C([H])([H])OC([H])([H])[N+]2=C([H])C([H])=C(C([H])=C2[H])C([H])=NO[H]	14	-0.015	61	0.4175	0.402	66	0.833	1.510	54	3	2.003	40	4	2.174	2.169	288	2	5	2	2	6
TMB-4	[H]/C1=C(/C([H])=C([H])[N+](=C1[H])C([H])([H])C([H])([H])C([H])([H])[N+]2=C([H])C([H])=C(C([H])=C2[H])C([H])=NO[H])C([H])=NO[H]	12	0.431	65	0.335	1.217	85	2	1.161	43	2	2.258	62	6	2.403	2.397	286	2	4	2	2	6

Table S2. Individual principal component (PC) analysis on 115 compounds tested for reactivation of GB-inhibited BChE. List of loadings for 18 variables ordered according to PC1. Proportion of variance is listed in the brackets with the cumulative proportion of variance for the selected components 82.2 %.

	Loading		
	PC1 (51.8 %)	PC2 (19.3 %)	PC3 (11.1 %)
Eigenvalue	9.32	3.52	2.0
Time, h (GB)	0.627	-0.239	-0.634
- log (k_{obs} min^{-1}) (GB)	0.566	-0.172	-0.755
Molecular fractional polar surface area	0.252	-0.527	-0.303
ADMET Alog*P*98	-0.244	0.916	-0.226
Alog*P*	-0.429	0.856	-0.154
log*D*	-0.433	0.855	-0.159
Dipole magnitude	-0.505	0.190	-0.107
Oxime inhibition,	-0.578	0.173	-0.293
Number of H bond donor atoms	-0.614	-0.574	-0.019
React$_{max}$, % (GB)	-0.642	0.044	0.671
Number of H bond acceptor atoms	-0.847	-0.398	-0.147
ADMET PSA 2D	-0.868	-0.434	-0.071
Number of rings	-0.882	0.049	-0.362
Number of aromatic rings	-0.893	0.061	-0.334
Number of rotational bonds	-0.932	-0.159	0.078
Molecular surface area	-0.964	-0.186	-0.014
Molecular volume	-0.970	-0.139	-0.058
Molecular weight	-0.974	-0.139	-0.110

Table S3. Individual principal component (PC) analysis on 115 compounds tested for reactivation of GF-inhibited BChE. List of loadings for 18 variables ordered according to PC1. Proportion of variance is listed in the brackets with the cumulative proportion of variance for the selected components 79.3 %.

	Loadings		
	PC1 (49.9 %)	PC2 (19.7 %)	PC3 (9.7 %)
Eigenvalue	8.99	3.54	1.75
- log (k_{obs} min^{-1}) (GF)	0.557	0.275	-0.622
Time, h (GF)	0.446	0.209	-0.680
Molecular fractional polar surface area	0.202	-0.492	-0.353
ADMET Alog*P*98	-0.198	0.948	0.020
Alog*P*	-0.377	0.884	0.034
log*D*	-0.381	0.885	0.025
React$_{max}$,% (GF)	-0.439	-0.060	0.695
Dipole magnitude	-0.505	0.235	0.064
Oxime inhibition, %	-0.590	0.247	-0.076
Number of H bond donor atoms	-0.641	-0.516	-0.195
Number of H bond acceptor atoms	-0.876	-0.315	-0.178
Number of rings	-0.890	0.174	-0.301
ADMET PSA 2D	-0.890	-0.363	-0.158
Number of aromatic rings	-0.898	0.182	-0.279
Number of rotational bonds	-0.936	-0.115	0.076
Molecular surface area	-0.973	-0.119	-0.021
Molecular volume	-0.978	-0.064	-0.051
Molecular weight	-0.983	-0.054	-0.104

Table S4. Individual principal component (PC) analysis on 115 compounds tested for reactivation of VX-inhibited BChE. List of loadings for 18 variables ordered according to PC1. Proportion of variance is listed in the brackets with the cumulative proportion of variance for the selected components 82.1 %

	Loading		
	PC1 (50.3 %)	PC2 (19.3 %)	PC3 (12.5 %)
Eigenvalue	11.46	4.18	3.82
Time, h (VX)	0.487	0.152	-0.797
- log (k_{obs} min^{-1}) (VX)	0.481	0.120	-0.793
Molecular fractional polar surface area	0.242	-0.465	-0.512
ADMET AlogP98	-0.206	0.950	-0.014
Alog*P*	-0.390	0.884	0.022
log*D*	-0.394	0.883	0.022
Dipole magnitude	-0.487	0.240	-0.143
Oxime inhibition,	-0.567	0.258	-0.255
React$_{max}$, % (VX)	-0.590	-0.110	0.612
Number of H bond donor atoms	-0.642	-0.536	-0.107
Number of H bond acceptor atoms	-0.859	-0.321	-0.243
ADMET PSA 2D	-0.883	-0.374	-0.172
Number of rings	-0.888	0.157	-0.291
Number of aromatic rings	-0.898	0.163	-0.261
Number of rotational bonds	-0.936	-0.129	0.048
Molecular surface area	-0.973	-0.136	-0.027
Molecular volume	-0.979	-0.082	-0.053
Molecular weight	-0.983	-0.072	-0.102

CC BY

Table S5. Individual principal component (PC) analysis on 115 compounds tested for reactivation of GA-inhibited BChE. List of loadings for 18 variables ordered according to PC1. Proportion of variance is listed in the brackets with the cumulative proportion of variance for the selected components 78.5 %.

	Loadings		
	PC1 (47.0%)	PC2 (21.4%)	PC3 (10.1%)
Eigenvalue	8.46	3.85	1.83
Molecular fractional polar surface area	0.210	-0.512	0.002
- log (k_{obs} min^{-1}) (GA)	0.188	-0.490	-0.729
Time, h (GA)	0.001	-0.459	-0.521
React$_{max}$, % (GA)	-0.139	0.493	0.669
ADMET Alog*P*98	-0.249	0.886	-0.313
Alog*P*	-0.422	0.836	-0.242
log*D*	-0.427	0.835	-0.245
Dipole magnitude	-0.516	0.236	0.112
Oxime inhibition, %	-0.595	0.090	-0.488
Number of H bond donor atoms	-0.626	-0.524	0.204
Number of H bond acceptor atoms	-0.865	-0.370	0.051
ADMET PSA 2D	-0.877	-0.398	0.124
Number of rotational bonds	-0.919	-0.132	0.105
Number of rings	-0.922	0.061	-0.129
Number of aromatic rings	-0.928	0.073	-0.119
Molecular surface area	-0.964	-0.161	0.071
Molecular volume	-0.976	-0.113	0.050
Molecular weight	-0.985	-0.116	0.022

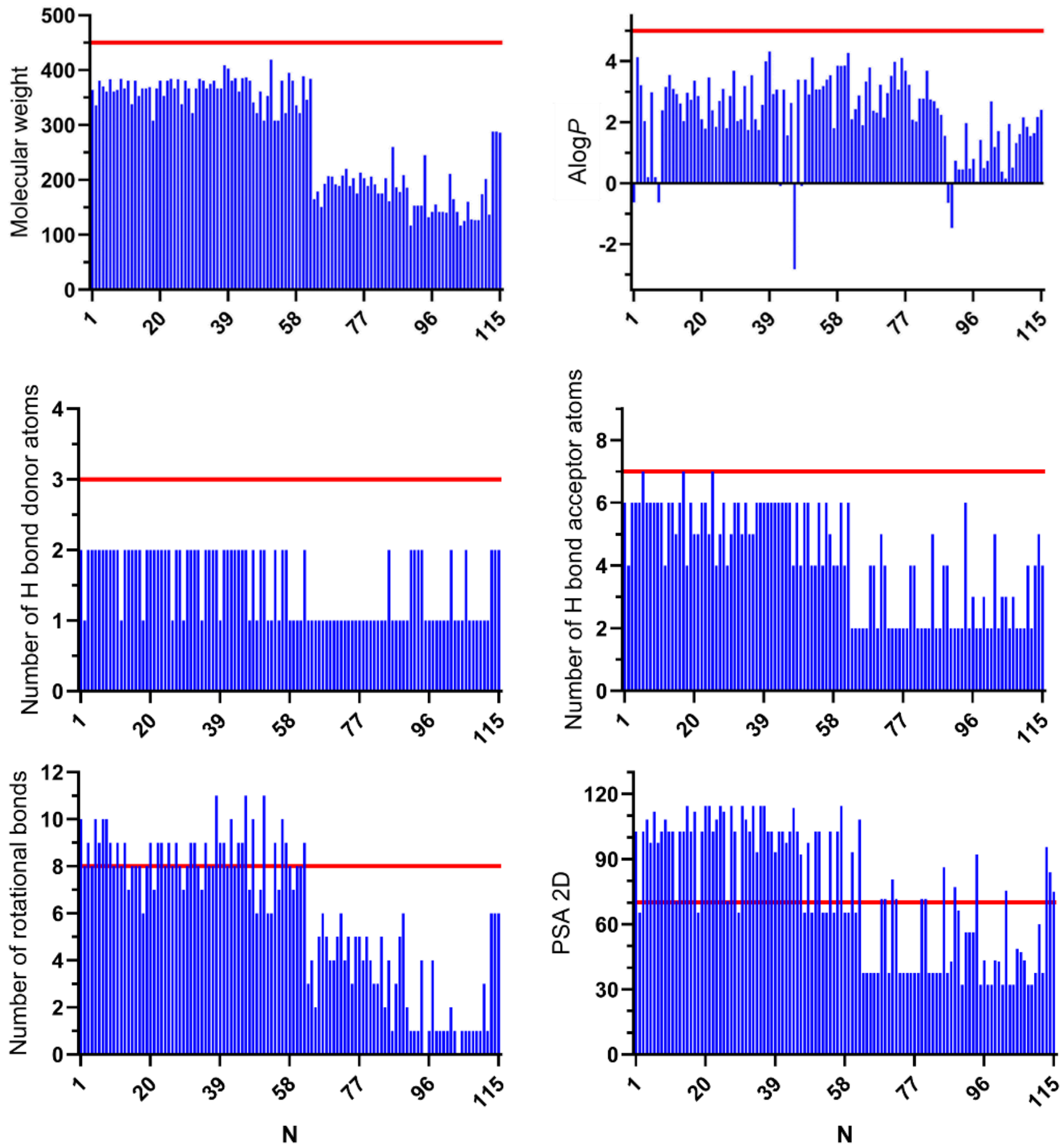

Figure S2. Plot of physicochemical properties of the studied oximes (N) (Table S1). Values below the red line represent recommended values for the CNS-active drugs [1]

Reference

[1] H. Pajouhesh, G.R. Lenz. Medicinal Chemical Properties of Successful Central Nervous System Drugs. *NeuroRX* **2** (2005) 541-553, https://doi.org/10.1602/neurorx.2.4.541
