# Supplementary material for: Assessing oxime reactivation efficacy using principal component analysis: Insights from nerve agents inhibited human butyrylcholinesterase
Source: ADMET DMPK. 2026 May 28;14:3361. doi: 10.5599/admet.3361 (PMC13499654; doi:10.5599/admet.3361)
Supplement: Supplementary file 2 [file ADMET-14-3361-S1.docx]

ADMET & DMPK **14** (2026) S3361

**Open Access : ISSN : 1848-7718**
[**http://www.pub.iapchem.org/ojs/index.php/admet**](http://www.pub.iapchem.org/ojs/index.php/admet)

Supplementary material to

Assessing oxime reactivation efficacy using principal component analysis: Insights from nerve agents inhibited human butyrylcholinesterase

Goran Šinko^1,^^<https://orcid.org/0000-0002-8265-1901>^, Tena Čadež^1^^<https://orcid.org/0000-0002-1301-6816>^, Zrinka Kovarik^1,2^^<https://orcid.org/0000-0001-9863-886X>^ and Nikolina Maček Hrvat^1^^<https://orcid.org/0000-0003-1682-8999>^

ADMET & DMPK **14** (2026) 3361; <https://doi.org/10.5599/admet.3361>

**Figure S1.** Plot of three-dimensional data projected into the new coordinate system of principal components is available at <https://pub.iapchem.org/ojs/SINKO_PCA_PLOT-3D_scores.html>

**Table S1.** Kinetic data for reactivation of BChE inhibited by organophosphorus compounds sarin (GB), cyclorasin (GF), VX and tabun (GA) together with ADME data for a library of 115 oximes including including click-chemistry-derived ones

| NAME | SMILES | Oxime inhibition, % | - log (*k*_obs_ / min^-1^) (GB) | React_max_, % GB | Time, h (GB) | - log (*k*_obs_ /min^-1^) (GF) | React_max_, % (GF) | Time, h (GF) | - log (*k*_obs_ /min^-1^) (VX) | React_max_, % (VX) | Time, h (VX) | - log (*k*_obs_ / min^-1^) (GA) | React_max_, % (GA) | Time, h (GA) | A log*P* | log*D* | Molecular weight | Number of aromatic rings | Number of H bond acceptor atoms | Number of H bond donor atoms | Number of rings | Number of rotational bonds |
| --- | --- | --- | --- | --- | --- | --- | --- | --- | --- | --- | --- | --- | --- | --- | --- | --- | --- | --- | --- | --- | --- | --- |
| 1A | [H]C=1N(N=NC=1C([H])([H])C([H])([H])[N+]=2C([H])=C([H])N(C=2C([H])=NO[H])C([H])([H])[H])C([H])([H])C([H])([H])C([H])([H])[N+](C([H])([H])C([H])=NO[H])(C([H])([H])[H])C([H])([H])[H] | 46 | 1.609 | 86 | 4.5 | 0.529 | 93 | 0.5 | 1.393 | 57 | 2 | 5.000 | 20 | 24 | -0.62 | -0.7 | 364 | 2 | 6 | 2 | 2 | 10 |
| 2A | [H]/C1=C(/[N+](=C(/[H])C([H])=C1[H])C([H])([H])C([H])([H])C([H])([H])C([H])([H])C=2N=NN(C=2[H])C([H])([H])C3=C([H])C([H])=C([H])C([H])=C3[H])C([H])=NO[H] | 36 | 2.131 | 81 | 10 | 1.381 | 80 | 2.25 | 1.959 | 61 | 6.3 | 2.292 | 66 | 10 | 4.142 | 4.102 | 336 | 3 | 4 | 1 | 3 | 8 |
| 3A | [H]C=1C([H])=C([H])[N+](=C(C=1[H])C([H])=NO[H])C([H])([H])C([H])([H])C=2N=NN(C=2[H])C([H])([H])C([H])([H])C([H])([H])[N+]3=C([H])C([H])=C([H])C([H])=C3C([H])=NO[H] | 52 | 1.061 | 81 | 1.75 | 0.201 | 70 | 0.17 | 1.218 | 60 | 1.3 | 2.033 | 27 | 6 | 3.215 | 3.158 | 381 | 3 | 6 | 2 | 3 | 9 |
| 4A | [H]/C1=[N+](/C([H])=C([H])C(=C1[H])C([H])=NO[H])C([H])([H])C([H])([H])N2N=N/C(=C2/[H])C([H])([H])C([H])([H])[N+]3=C(N(C([H])=C3[H])C([H])([H])[H])C([H])=NO[H] | 22 | 0.745 | 91 | 0.5 | 0.285 | 73 | 0.28 | 1.146 | 63 | 1 | 2.370 | 32 | 24 | 2.039 | 2.02 | 370 | 3 | 6 | 2 | 3 | 8 |
| 5A | [H]C=1N(N=NC=1C([H])([H])C([H])([H])[N+]2=C([H])C([H])=C([H])C([H])=C2C([H])=NO[H])C([H])([H])C([H])([H])C([H])([H])[N+](C([H])([H])C([H])=NO[H])(C([H])([H])[H])C([H])([H])[H] | 25 | 0.945 | 71 | 1 | 0.407 | 77 | 0.5 | 1.320 | 65 | 2 | 5.000 | 20 | 24 | 0.199 | 0.141 | 361 | 2 | 6 | 2 | 2 | 10 |
| 6A | [H]C=1N(N=NC=1C([H])([H])C([H])([H])[N+]2=C([H])C([H])=C([H])C([H])=C2C([H])=NO[H])C([H])([H])OC([H])([H])[N+]3=C(C([H])=C([H])C([H])=C3[H])C([H])=NO[H] | 56 | 0.027 | 69 | 0.065 | 1.340 | 16 | 1 | 0.971 | 46 | 3.5 | 5.000 | 20 | 24 | 2.987 | 2.919 | 383 | 3 | 7 | 2 | 3 | 9 |
| 7A | [H]C=1N(N=NC=1C([H])([H])C([H])([H])[N+](C([H])([H])[H])(C([H])([H])C([H])=NO[H])C([H])([H])[H])C([H])([H])C([H])([H])C([H])([H])[N+]2=C(C([H])=C([H])C([H])=C2[H])C([H])=NO[H] | 22 | 0.884 | 94 | 1 | 0.236 | 67 | 0.17 | 1.340 | 54 | 1.3 | 5.000 | 20 | 24 | 0.199 | 0.158 | 361 | 2 | 6 | 2 | 2 | 10 |
| 8A | [H]C=1N(N=NC=1C([H])([H])C([H])([H])[N+](C([H])([H])[H])(C([H])([H])C([H])=NO[H])C([H])([H])[H])C([H])([H])C([H])([H])C([H])([H])[N+]2=C(N(C([H])=C2[H])C([H])([H])[H])C([H])=NO[H] | 24 | 1.513 | 72 | 2.5 | 1.020 | 74 | 3.5 | 1.561 | 49 | 2 | 5.000 | 20 | 24 | -0.62 | -0.69 | 364 | 2 | 6 | 2 | 2 | 10 |
| 9A | [H]C=1N(N=NC=1C([H])([H])C([H])([H])[N+]=2C([H])=C([H])N(C=2C([H])=NO[H])C([H])([H])[H])C([H])([H])C([H])([H])C([H])([H])[N+]3=C(C([H])=C([H])C([H])=C3[H])C([H])=NO[H] | 49 | 1.642 | 82 | 4 | 0.346 | 64 | 0.25 | 1.384 | 55 | 1.5 | 5.000 | 20 | 24 | 2.393 | 2.32 | 384 | 3 | 6 | 2 | 3 | 9 |
| 10A | [H]/C1=C(\[H])[N+](=C(C([H])=C1[H])C([H])=NO[H])C([H])([H])C([H])([H])N2N=N/C(=C2/[H])C([H])([H])C([H])([H])[N+]3=C([H])C([H])=C([H])C([H])=C3C([H])=NO[H] | 39 | 1.027 | 84 | 1.75 | 0.263 | 83 | 0.25 | 1.214 | 58 | 1 | 2.407 | 24 | 24 | 3.153 | 3.135 | 367 | 3 | 6 | 2 | 3 | 8 |
| 11A | [H]/C1=C(\[H])[N+](=C(C([H])=C1[H])C([H])=NO[H])C([H])([H])C([H])([H])C([H])([H])C([H])([H])N2N=N/C(=C2/[H])C([H])([H])[N+]3=C([H])C([H])=C([H])C([H])=C3C([H])=NO[H] | 72 | 1.375 | 81 | 2 | 0.754 | 66 | 0.75 | 1.593 | 67 | 14 | 5.000 | 20 | 24 | 3.547 | 3.458 | 381 | 3 | 6 | 2 | 3 | 9 |
| 12A | [H]C=1N(N=NC=1C([H])([H])C([H])([H])[N+]2=C([H])C([H])=C([H])C([H])=C2C([H])=NO[H])C([H])([H])C([H])([H])C([H])([H])[N+]3=C([H])C([H])=C([H])C([H])=C3[H] | 39 | 1.134 | 83 | 1.5 | 0.498 | 58 | 0.17 | 1.611 | 66 | 5.5 | 5.000 | 20 | 24 | 3.097 | 3.04 | 338 | 3 | 4 | 1 | 3 | 8 |
| 13A | [H]C=1N(N=NC=1C([H])([H])C([H])([H])[N+]2=C([H])C([H])=C([H])C([H])=C2C([H])=NO[H])C([H])([H])C([H])([H])C([H])([H])[N+]3=C([H])C([H])=C(C([H])=C3[H])C([H])=NO[H] | 30 | 0.628 | 86 | 0.5 | -0.095 | 85 | 0.08 | 0.951 | 67 | 0.8 | 2.652 | 54 | 24 | 2.924 | 2.867 | 381 | 3 | 6 | 2 | 3 | 9 |
| 14A | [H]/C1=C(/N=NN1C([H])([H])C([H])([H])[N+]=2C([H])=C([H])C(=C([H])C=2[H])C([H])=NO[H])C([H])([H])[N+]3=C([H])C([H])=C([H])C([H])=C3C([H])=NO[H] | 31 | 0.825 | 87 | 0.875 | 0.145 | 52 | 0.08 | 1.034 | 46 | 0.8 | 2.521 | 27 | 24 | 2.613 | 2.597 | 353 | 3 | 6 | 2 | 3 | 7 |
| 15A | O=C(C=1C([H])=C([H])[N+](=C([H])C=1[H])C([H])([H])C([H])([H])N2N=N/C(=C2/[H])C([H])([H])C([H])([H])[N+]3=C([H])C([H])=C([H])C([H])=C3C([H])=NO[H])N([H])[H] | 19 | 1.134 | 49 | 0.25 | 0.400 | 68 | 0.5 | 1.417 | 93 | 2 | 2.706 | 27 | 24 | 2.037 | 2.019 | 367 | 3 | 5 | 2 | 3 | 8 |
| 16A | [H]C=1N(N=NC=1C([H])([H])[N+]2=C([H])C([H])=C([H])C([H])=C2C([H])=NO[H])C([H])([H])C([H])([H])C([H])([H])[N+]3=C(C([H])=C([H])C([H])=C3[H])C([H])=NO[H] | 79 | 1.137 | 77 | 2 | 0.332 | 81 | 0.33 | 1.516 | 68 | 2.5 | 5.000 | 20 | 24 | 2.967 | 2.949 | 367 | 3 | 6 | 2 | 3 | 8 |
| 17A | [H]C=1N(N=NC=1C([H])([H])[N+]2=C([H])C([H])=C([H])C([H])=C2C([H])=NO[H])C([H])([H])OC([H])([H])[N+]3=C(C([H])=C([H])C([H])=C3[H])C([H])=NO[H] | 62 | 0.841 | 71 | 1 | 0.374 | 58 | 0.33 | 1.295 | 55 | 2 | 5.000 | 20 | 24 | 2.738 | 2.708 | 369 | 3 | 7 | 2 | 3 | 8 |
| 18A | [H]/C1=C(/[N+](=C(/[H])C([H])=C1[H])C([H])([H])C([H])([H])C=2N=NN(C=2[H])C([H])([H])C3=C([H])C([H])=C([H])C([H])=C3[H])C([H])=NO[H] | 51 | 2.301 | 63 | 17 | 0.413 | 65 | 1.75 | 2.658 | 55 | 24 | 5.000 | 20 | 24 | 3.365 | 3.347 | 308 | 3 | 4 | 1 | 3 | 6 |
| 19A | [H]/C1=C(\[H])[N+](=C([H])C([H])=C1C([H])=NO[H])C([H])([H])C([H])([H])N2N=N/C(=C2/[H])C([H])([H])C([H])([H])[N+]3=C([H])C([H])=C([H])C([H])=C3C([H])=NO[H] | 25 | 0.757 | 84 | 0.75 | -0.167 | 52 | 0.08 | 1.187 | 83 | 2 | 2.545 | 35 | 24 | 2.861 | 2.844 | 367 | 3 | 6 | 2 | 3 | 8 |
| 20A | O=C(C=1C([H])=C([H])[N+](=C([H])C=1[H])C([H])([H])C([H])([H])C([H])([H])N2N=N/C(=C2/[H])C([H])([H])C([H])([H])[N+]3=C([H])C([H])=C([H])C([H])=C3C([H])=NO[H])N([H])[H] | 29 | 1.047 | 79 | 1 | 0.465 | 75 | 0.5 | 1.439 | 67 | 2 | 5.000 | 20 | 24 | 2.099 | 2.042 | 381 | 3 | 5 | 2 | 3 | 9 |
| 21A | O=C(C=1C([H])=C([H])[N+](=C([H])C=1[H])C([H])([H])C([H])([H])N2N=N/C(=C2/[H])C([H])([H])[N+]3=C([H])C([H])=C([H])C([H])=C3C([H])=NO[H])N([H])[H] | 28 | 1.148 | 85 | 1 | 0.730 | 76 | 0.5 | 1.650 | 67 | 2.5 | 5.000 | 20 | 24 | 1.789 | 1.773 | 353 | 3 | 5 | 2 | 3 | 7 |
| 22A | [H]/C1=C(\[H])[N+](=C(C([H])=C1[H])C([H])=NO[H])C([H])([H])C([H])([H])N2N=N/C(=C2/[H])C([H])([H])C([H])([H])C([H])([H])[N+]3=C([H])C([H])=C([H])C([H])=C3C([H])=NO[H] | 59 | 1.627 | 87 | 3.5 | 0.616 | 81 | 0.75 | 1.666 | 60 | 3.5 | 2.527 | 29 | 24 | 3.474 | 3.422 | 381 | 3 | 6 | 2 | 3 | 9 |
| 23A | [H]C=1N(N=NC=1C([H])([H])C([H])([H])[N+]2=C([H])C([H])=C([H])C([H])=C2C([H])=NO[H])C([H])([H])C([H])([H])C([H])([H])[N+]3=C(N(C([H])=C3[H])C([H])([H])[H])C([H])=NO[H] | 52 | 1.633 | 86 | 5 | 0.112 | 57 | 0.17 | 1.688 | 52 | 2.3 | 2.435 | 40 | 24 | 2.393 | 2.322 | 384 | 3 | 6 | 2 | 3 | 9 |
| 24A | O=C(/C1=C(\[H])C([H])=[N+](C([H])=C1[H])C([H])([H])C([H])([H])C([H])([H])N2N=N/C(=C2/[H])C([H])([H])[N+]=3C([H])=C([H])C([H])=C([H])C=3C([H])=NO[H])N([H])[H] | 53 | 1.613 | 80 | 4 | 0.696 | 58 | 0.5 | 2.027 | 57 | 3.5 | 5.000 | 20 | 24 | 1.851 | 1.835 | 367 | 3 | 5 | 2 | 3 | 8 |
| 25A | [H]C=1N(N=NC=1C([H])([H])C([H])([H])[N+]2=C([H])C([H])=C(C([H])=C2[H])C([H])=NO[H])C([H])([H])OC([H])([H])[N+]3=C([H])C([H])=C([H])C([H])=C3C([H])=NO[H] | 42 | 1.309 | 64 | 2 | 0.342 | 70 | 0.33 | 1.907 | 57 | 7.5 | 5.000 | 20 | 24 | 2.695 | 2.627 | 383 | 3 | 7 | 2 | 3 | 9 |
| 26A | [H]C=1N(N=NC=1C([H])([H])C([H])([H])[N+]2=C([H])C([H])=C([H])C([H])=C2[H])C([H])([H])C([H])([H])C([H])([H])[N+]3=C([H])C([H])=C([H])C([H])=C3C([H])=NO[H] | 49 | 1.123 | 87 | 1.5 | 0.794 | 62 | 0.5 | 1.439 | 82 | 3 | 5.000 | 20 | 24 | 3.097 | 3.056 | 338 | 3 | 4 | 1 | 3 | 8 |
| 27A | O=C(C=1C([H])=C([H])[N+](=C([H])C=1[H])C([H])([H])C([H])([H])C([H])([H])N2N=N/C(=C2/[H])C([H])([H])C([H])([H])[N+]3=C([H])C([H])=C(C([H])=C3[H])C([H])=NO[H])N([H])[H] | 27 | 0.963 | 82 | 1 | -0.211 | 55 | 0.08 | 1.429 | 60 | 2 | 2.636 | 41 | 24 | 1.808 | 1.805 | 381 | 3 | 5 | 2 | 3 | 9 |
| 28A | [H]/C1=C(\[H])[N+](=C(C([H])=C1[H])C([H])=NO[H])C([H])([H])C([H])([H])N2N=N/C(=C2/[H])C([H])([H])C([H])([H])[N+]3=C([H])C([H])=C(C([H])=C3[H])C([H])=NO[H] | 24 | 0.915 | 92 | 1 | -0.045 | 80 | 0.08 | 1.192 | 54 | 1.5 | 2.400 | 20 | 24 | 2.861 | 2.845 | 367 | 3 | 6 | 2 | 3 | 8 |
| 29A | [H]/C1=C(/[N+](=C(/[H])C([H])=C1[H])C([H])([H])C([H])([H])C([H])([H])C=2N=NN(C=2[H])C([H])([H])C3=C([H])C([H])=C([H])C([H])=C3[H])C([H])=NO[H] | 35 | 2.301 | 72 | 24 | 1.405 | 73 | 2.25 | 2.585 | 52 | 24 | 2.251 | 66 | 10 | 3.686 | 3.668 | 322 | 3 | 4 | 1 | 3 | 7 |
| 30A | O=C(/C1=C(\[H])C([H])=[N+](C([H])=C1[H])C([H])([H])C([H])([H])C=2N=NN(C=2[H])C([H])([H])C([H])([H])[N+]=3C([H])=C([H])C([H])=C([H])C=3C([H])=NO[H])N([H])[H] | 24 | 1.130 | 85 | 1.25 | 0.598 | 65 | 0.5 | 1.635 | 76 | 2.3 | 2.614 | 22 | 24 | 2.037 | 2.021 | 367 | 3 | 5 | 2 | 3 | 8 |
| 31A | [H]C=1N(N=NC=1C([H])([H])C([H])([H])[N+]=2C([H])=C([H])N(C=2C([H])=NO[H])C([H])([H])[H])C([H])([H])C([H])([H])C([H])([H])[N+]3=C([H])C([H])=C(C([H])=C3[H])C([H])=NO[H] | 46 | 0.866 | 83 | 1.5 | 0.075 | 70 | 0.17 | 0.964 | 64 | 1.5 | 2.793 | 48 | 24 | 2.101 | 2.029 | 384 | 3 | 6 | 2 | 3 | 9 |
| 32A | [H]C([H])([N+]=1C([H])=C([H])C([H])=C([H])C=1C([H])=NO[H])C([H])([H])N2N=N/C(=C2/[H])C([H])([H])C([H])([H])C([H])([H])[N+]3=C([H])C([H])=C(C([H])=C3[H])C([H])=NO[H] | 39 | 0.781 | 86 | 0.625 | 0.323 | 92 | 0.33 | 1.166 | 67 | 2 | 2.793 | 31 | 24 | 3.183 | 3.132 | 381 | 3 | 6 | 2 | 3 | 9 |
| 33A | O=C(/C1=C(\[H])C([H])=[N+](C([H])=C1[H])C([H])([H])C([H])([H])C=2N=NN(C=2[H])C([H])([H])C([H])([H])[N+]=3C([H])=C([H])C(=C([H])C=3[H])C([H])=NO[H])N([H])[H] | 17 | 1.194 | 79 | 1.25 | 0.157 | 53 | 0.17 | 1.264 | 54 | 1.3 | 2.523 | 30 | 24 | 1.746 | 1.743 | 367 | 3 | 5 | 2 | 3 | 8 |
| 34A | [H]/C1=[N+](/C(=C(/[H])C([H])=C1[H])C([H])=NO[H])C([H])([H])C([H])([H])C=2N=NN(C=2[H])C([H])([H])N3N=N/C(=C3/[H])C4=C([H])C([H])=C([H])C([H])=C4[H] | 17 | 2.921 | 58 | 14.5 | 2.252 | 63 | 17 | 2.569 | 33 | 24 | 5.000 | 20 | 24 | 3.542 | 3.524 | 375 | 4 | 6 | 1 | 4 | 7 |
| 35A | O=C(/C1=C(\[H])C([H])=[N+](C([H])=C1[H])C([H])([H])C([H])([H])C=2N=NN(C=2[H])C([H])([H])C([H])([H])C([H])([H])[N+]3=C([H])C([H])=C([H])C([H])=C3C([H])=NO[H])N([H])[H] | 27 | 1.241 | 86 | 1.75 | 0.588 | 60 | 0.5 | 1.747 | 75 | 2.5 | 5.000 | 20 | 24 | 2.099 | 2.059 | 381 | 3 | 5 | 2 | 3 | 9 |
| 36A | O=C(C=1C([H])=C([H])[N+](=C([H])C=1[H])C([H])([H])C([H])([H])N2N=N/C(=C2/[H])C([H])([H])C([H])([H])[N+]3=C([H])C([H])=C(C([H])=C3[H])C([H])=NO[H])N([H])[H] | 19 | 1.044 | 87 | 1 | 0.245 | 55 | 0.17 | 1.268 | 60 | 1.5 | 2.721 | 44 | 24 | 1.746 | 1.743 | 367 | 3 | 5 | 2 | 3 | 8 |
| 37A | [H]/C1=C(\[H])[N+](=C([H])C([H])=C1C([H])=NO[H])C([H])([H])C([H])([H])N2N=N/C(=C2/[H])C([H])([H])C([H])([H])[N+]3=C([H])C([H])=C(C([H])=C3[H])C([H])=NO[H] | 15 | 0.573 | 84 | 0.5 | 0.057 | 57 | 0.17 | 1.093 | 76 | 1.3 | 2.717 | 53 | 24 | 2.57 | 2.567 | 367 | 3 | 6 | 2 | 3 | 8 |
| 38A | [H]/C1=C(\[H])[N+](=C(C([H])=C1[H])C([H])=NO[H])C([H])([H])C([H])([H])C([H])([H])C([H])([H])C=2N=NN(C=2[H])C([H])([H])C([H])([H])C([H])([H])[N+]3=C(C([H])=C([H])C([H])=C3[H])C([H])=NO[H] | 68 | 1.287 | 88 | 2.25 | 0.234 | 75 | 0.17 | 1.775 | 57 | 3.5 | 5.000 | 20 | 24 | 3.993 | 3.952 | 409 | 3 | 6 | 2 | 3 | 11 |
| 39A | [H]/C1=[N+](/C(=C(/[H])C([H])=C1[H])C([H])=NO[H])C([H])([H])C([H])([H])C([H])([H])C([H])([H])C=2N=NN(C=2[H])C([H])([H])N3N=N/C(=C3/[H])C4=C([H])C([H])=C([H])C([H])=C4[H] | 26 | 5.000 | 20 | 14.5 | 2.347 | 64 | 24 | 5.000 | 20 | 24 | 2.317 | 71 | 19.3 | 4.319 | 4.279 | 403 | 4 | 6 | 1 | 4 | 9 |
| 40A | [H]C=1N(N=NC=1C([H])([H])C([H])([H])[N+]2=C([H])C([H])=C(C([H])=C2[H])C([H])=NO[H])C([H])([H])C([H])([H])C([H])([H])[N+]3=C([H])C([H])=C([H])C([H])=C3C([H])=NO[H] | 28 | 0.860 | 93 | 0.875 | 0.135 | 70 | 0.17 | 1.350 | 65 | 2.2 | 5.000 | 20 | 24 | 2.924 | 2.883 | 381 | 3 | 6 | 2 | 3 | 9 |
| 41A | [F]C=1C([H])=[N+](C([H])=C([H])C=1C([H])=NO[H])C([H])([H])C([H])([H])N2N=N/C(=C2/[H])C([H])([H])C([H])([H])[N+]3=C([H])C([H])=C([H])C([H])=C3C([H])=NO[H] | 27 | 1.349 | 89 | 1.75 | 0.504 | 64 | 0.5 | 1.611 | 65 | 2.5 | 2.697 | 28 | 24 | 3.067 | 3.049 | 385 | 3 | 6 | 2 | 3 | 8 |
| 42A | [H]C=1N(N=NC=1C([H])([H])C([H])([H])[N+](C([H])([H])[H])(C([H])([H])C([H])=NO[H])C([H])([H])[H])C([H])([H])C([H])([H])C([H])([H])[N+]2=C([H])C([H])=C(C([H])=C2[H])C([H])=NO[H] | 21 | 0.853 | 92 | 1 | 0.915 | 66 | 1 | 1.085 | 67 | 1.5 | 2.550 | 53 | 24 | -0.09 | -0.1 | 361 | 2 | 6 | 2 | 2 | 10 |
| 43A | [F]C=1/C(=C(/[H])C([H])=[N+](C=1[H])C([H])([H])C([H])([H])C=2N=NN(C=2[H])C([H])([H])C([H])([H])[N+]=3C([H])=C([H])C([H])=C([H])C=3C([H])=NO[H])C([H])=NO[H] | 25 | 0.940 | 84 | 0.875 | 0.298 | 74 | 0.17 | 1.330 | 62 | 1.8 | 5.000 | 20 | 24 | 3.067 | 3.051 | 385 | 3 | 6 | 2 | 3 | 8 |
| 44A | [H]C=1N(N=NC=1C([H])([H])C([H])([H])[N+]=2C([H])=C([H])N(C=2C([H])=NO[H])C([H])([H])[H])C([H])([H])C([H])([H])C([H])([H])[N+]3=C(N(C([H])=C3[H])C([H])([H])[H])C([H])=NO[H] | 60 | 1.903 | 83 | 6 | 0.564 | 67 | 0.625 | 1.896 | 60 | 4 | 5.000 | 20 | 24 | 1.57 | 1.498 | 387 | 3 | 6 | 2 | 3 | 9 |
| 45A | [H]C=1N(N=NC=1C([H])([H])C([H])([H])[N+]2=C([H])C([H])=C(C([H])=C2[H])C([H])=NO[H])C([H])([H])C([H])([H])C([H])([H])[N+]3=C([H])C([H])=C(C([H])=C3[H])C([H])=NO[H] | 17 | 0.306 | 90 | 0.17 | -0.133 | 88 | 0.08 | 0.959 | 55 | 1 | 2.903 | 46 | 24 | 2.632 | 2.63 | 381 | 3 | 6 | 2 | 3 | 9 |
| 46A | [H]C=1N(N=NC=1C([H])([H])C([H])([H])[N+](C([H])([H])[H])(C([H])([H])C([H])=NO[H])C([H])([H])[H])C([H])([H])C([H])([H])C([H])([H])[N+](C([H])([H])C([H])=NO[H])(C([H])([H])[H])C([H])([H])[H] | 19 | 2.585 | 89 | 24 | 0.886 | 64 | 1 | 1.618 | 55 | 3 | 5.000 | 20 | 24 | -2.82 | -2.82 | 341 | 1 | 6 | 2 | 1 | 11 |
| 47A | [H]/C1=C(\[H])[N+](=C([H])C([H])=C1C([H])=NO[H])C([H])([H])C([H])([H])C([H])([H])C=2N=NN(C=2[H])C([H])([H])C3=C([H])C([H])=C([H])C([H])=C3[H] | 31 | 1.326 | 55 | 2 | 1.135 | 55 | 1.875 | 1.530 | 48 | 3 | 2.609 | 20 | 24 | 3.395 | 3.392 | 322 | 3 | 4 | 1 | 3 | 7 |
| 48A | [H]C=1N(N=NC=1C([H])([H])C([H])([H])[N+]2=C([H])C([H])=C(C([H])=C2[H])C([H])=NO[H])C([H])([H])C([H])([H])C([H])([H])[N+](C([H])([H])C([H])=NO[H])(C([H])([H])[H])C([H])([H])[H] | 19 | 1.093 | 75 | 1 | 0.214 | 79 | 0.25 | 1.265 | 63 | 3.3 | 5.000 | 20 | 24 | -0.09 | -0.1 | 361 | 2 | 6 | 2 | 2 | 10 |
| 49A | [H]/C1=C(\[H])[N+](=C(C([H])=C1[H])C([H])=NO[H])C([H])([H])C([H])([H])C([H])([H])N2N=N/C(=C2/[H])C3=C([H])C([H])=C([H])C([H])=C3[H] | 86 | 5.000 | 20 | 14 | 1.754 | 45 | 14.5 | 5.000 | 20 | 24 | 5.000 | 20 | 24 | 3.393 | 3.375 | 308 | 3 | 4 | 1 | 3 | 6 |
| 50A | [H]C([H])([N+]=1C([H])=C([H])C([H])=C([H])C=1C([H])=NO[H])C([H])([H])N2N=N/C(=C2/[H])C([H])([H])[N+]3=C([H])C([H])=C([H])C([H])=C3C([H])=NO[H] | 48 | 0.877 | 82 | 1.5 | 0.174 | 77 | 0.17 | 1.394 | 71 | 2 | 5.000 | 20 | 24 | 2.905 | 2.889 | 353 | 3 | 6 | 2 | 3 | 7 |
| 51A | [H]/C1=C(\[H])C([H])=C([H])C(=[N+]1C([H])([H])C([H])([H])C#CC([H])([H])C=2N=NN(C=2[H])C([H])([H])C([H])([H])C([H])([H])[N+]3=C([H])C([H])=C([H])C([H])=C3C([H])=NO[H])C([H])=NO[H] | 60 | 1.199 | 82 | 1.75 | 0.178 | 85 | 0.21 | 1.788 | 60 | 14.5 | 5.000 | 20 | 24 | 4.127 | 4.067 | 419 | 3 | 6 | 2 | 3 | 11 |
| 62A | [H]/C1=C(\[H])[N+](=C([H])C([H])=C1C([H])=NO[H])C([H])([H])C([H])([H])C=2N=NN(C=2[H])C([H])([H])C3=C([H])C([H])=C([H])C([H])=C3[H] | 25 | 0.819 | 69 | 1.5 | 1.360 | 70 | 2 | 1.460 | 53 | 2.3 | 5.000 | 20 | 24 | 3.074 | 3.071 | 308 | 3 | 4 | 1 | 3 | 6 |
| 53A | [H]/C1=C(/C([H])=C([H])C([H])=[N+]1C([H])([H])C([H])([H])C=2N=NN(C=2[H])C([H])([H])C3=C([H])C([H])=C([H])C([H])=C3[H])C([H])=NO[H] | 27 | 1.324 | 74 | 2 | 0.422 | 72 | 0.5 | 1.425 | 60 | 2.3 | 5.000 | 20 | 24 | 3.074 | 3.069 | 308 | 3 | 4 | 1 | 3 | 6 |
| 54A | [H]C([H])([N+]=1C([H])=C([H])C(=C([H])C=1[H])C([H])=NO[H])C([H])([H])N2N=N/C(=C2/[H])C([H])([H])C([H])([H])C([H])([H])[N+]3=C([H])C([H])=C([H])C([H])=C3C([H])=NO[H] | 33 | 0.759 | 81 | 0.75 | 0.451 | 59 | 0.17 | 1.293 | 73 | 1.8 | 2.332 | 21 | 24 | 3.183 | 3.143 | 381 | 3 | 6 | 2 | 3 | 9 |
| 55A | [H]/C1=C(/C([H])=C([H])C([H])=[N+]1C([H])([H])C([H])([H])C([H])([H])C=2N=NN(C=2[H])C([H])([H])C3=C([H])C([H])=C([H])C([H])=C3[H])C([H])=NO[H] | 42 | 1.224 | 70 | 2 | 0.536 | 63 | 0.625 | 1.474 | 56 | 2.8 | 2.360 | 38 | 2 | 3.395 | 3.39 | 322 | 3 | 4 | 1 | 3 | 7 |
| 56A | [H]/C1=C(\[H])[N+](=C(C([H])=C1[H])C([H])=NO[H])C([H])([H])C([H])([H])C([H])([H])C=2N=NN(C=2[H])C([H])([H])C([H])([H])C([H])([H])[N+]3=C([H])C([H])=C([H])C([H])=C3C([H])=NO[H] | 70 | 1.699 | 82 | 4 | 0.275 | 80 | 0.21 | 2.114 | 47 | 4.5 | 5.000 | 20 | 24 | 3.536 | 3.496 | 395 | 3 | 6 | 2 | 3 | 10 |
| 57A | O=C(/C1=C(\[H])C([H])=[N+](C([H])=C1[H])C([H])([H])C([H])([H])C=2N=NN(C=2[H])C([H])([H])C([H])([H])C([H])([H])[N+]3=C([H])C([H])=C(C([H])=C3[H])C([H])=NO[H])N([H])[H] | 20 | 0.816 | 78 | 0.625 | 0.184 | 69 | 0.17 | 1.051 | 66 | 1.1 | 2.491 | 43 | 24 | 1.808 | 1.805 | 381 | 3 | 5 | 2 | 3 | 9 |
| 58A | [H]/C1=C(\[H])[N+](=C([H])C([H])=C1C([H])=NO[H])C([H])([H])C([H])([H])C([H])([H])C([H])([H])C=2N=NN(C=2[H])C([H])([H])C3=C([H])C([H])=C([H])C([H])=C3[H] | 29 | 1.900 | 58 | 5.5 | 1.636 | 54 | 4.25 | 1.910 | 54 | 6.5 | 5.000 | 20 | 24 | 3.851 | 3.848 | 336 | 3 | 4 | 1 | 3 | 8 |
| 59A | [H]/C1=C(/C([H])=C([H])C([H])=[N+]1C([H])([H])C([H])([H])C([H])([H])C([H])([H])C=2N=NN(C=2[H])C3=C([H])C([H])=C([H])C([H])=C3[H])C([H])=NO[H] | 53 | 2.086 | 69 | 10 | 0.782 | 59 | 0.5 | 1.870 | 56 | 7 | 5.000 | 20 | 24 | 3.844 | 3.839 | 322 | 3 | 4 | 1 | 3 | 7 |
| 60A | [H]/C1=[N+](/C(=C(/[H])C([H])=C1[H])C([H])=NO[H])C([H])([H])C([H])([H])C([H])([H])C=2N=NN(C=2[H])C([H])([H])N3N=N/C(=C3/[H])C4=C([H])C([H])=C([H])C([H])=C4[H] | 30 | 2.367 | 74 | 9.5 | 1.719 | 61 | 1.75 | 5.000 | 20 | 24 | 2.498 | 39 | 24 | 3.863 | 3.845 | 389 | 4 | 6 | 1 | 4 | 8 |
| 61A | [H]/C1=C(\[H])[N+](=C(C([H])=C1[H])C([H])=NO[H])C([H])([H])C([H])([H])C#CC([H])([H])C=2N=NN(C=2[H])C([H])([H])C3=C([H])C([H])=C([H])C([H])=C3[H] | 56 | 2.721 | 50 | 12 | 2.409 | 69 | 24 | 5.000 | 20 | 24 | 5.000 | 20 | 24 | 4.277 | 4.216 | 346 | 3 | 4 | 1 | 3 | 8 |
| 62A | [H]C=1N(N=NC=1C([H])([H])C([H])([H])[N+]2=C([H])C([H])=C(C([H])=C2[H])C([H])=NO[H])C([H])([H])C([H])([H])C([H])([H])[N+]3=C(N(C([H])=C3[H])C([H])([H])[H])C([H])=NO[H] | 39 | 1.479 | 84 | 3.5 | 0.286 | 53 | 0.17 | 1.529 | 55 | 4.5 | 5.000 | 20 | 24 | 2.101 | 2.031 | 384 | 3 | 6 | 2 | 3 | 9 |
| 1B | [H]/C1=C(/[N+](=C(/[H])C([H])=C1[H])C([H])([H])C([H])([H])C([H])([H])[H])C([H])=NO[H] | 11 | 0.859 | 86 | 1.25 | 0.379 | 75 | 0.585 | 0.855 | 66 | 0.6 | 2.567 | 41 | 24 | 2.425 | 2.36 | 165 | 1 | 2 | 1 | 1 | 3 |
| 2B | [H]/C1=C(/[N+](=C(/[H])C([H])=C1[H])C([H])([H])C([H])([H])C([H])([H])C([H])([H])[H])C([H])=NO[H] | 13 | 0.320 | 81 | 0.21 | 0.440 | 79 | 0.25 | 1.221 | 59 | 0.7 | 2.620 | 36 | 24 | 2.881 | 2.821 | 179 | 1 | 2 | 1 | 1 | 4 |
| 3B | [H]/C1=C(/[N+](=C(/[H])C([H])=C1[H])C([H])([H])C([H])([H])[H])C([H])=NO[H] | 11 | 1.526 | 88 | 3 | 1.378 | 66 | 2.5 | 1.233 | 68 | 1.5 | 5.000 | 20 | 24 | 1.901 | 1.842 | 151 | 1 | 2 | 1 | 1 | 2 |
| 4B | [H]/C1=C(/[N+](=C(/[H])C([H])=C1[H])C([H])([H])C([H])([H])C([H])([H])C([H])([H])C([H])([H])[H])C([H])=NO[H] | 35 | 0.537 | 73 | 0.18 | -0.538 | 60 | 0.118 | 1.292 | 53 | 1.3 | 2.292 | 35 | 13 | 3.337 | 3.277 | 193 | 1 | 2 | 1 | 1 | 5 |
| 5B | [H]/C1=C(/[N+](=C(/[H])C([H])=C1[H])C([H])([H])C([H])([H])C([H])([H])C([H])([H])C([H])([H])C([H])([H])[H])C([H])=NO[H] | 37 | 0.801 | 85 | 0.5 | -0.456 | 79 | 0.033 | 0.454 | 65 | 0.2 | 2.210 | 44 | 6.7 | 3.793 | 3.733 | 207 | 1 | 2 | 1 | 1 | 6 |
| 1C | [N-]=[N+]=NC([H])([H])C([H])([H])C([H])([H])[N+]= 1/C(=C(/[H])C([H])=C([H])C=1[H])C([H])=NO[H] | 19 | 0.725 | 78 | 0.75 | 0.284 | 76 | 10 | 1.216 | 61 | 2 | 2.703 | 40 | 24 | 2.377 | 2.213 | 206 | 1 | 4 | 1 | 1 | 5 |
| 2C | [N-]=[N+]=NC([H])([H])C([H])([H])[N+]=1C([H])= C([H])C([H])=C([H])C=1C([H])=NO[H] | 29 | 1.369 | 82 | 2 | 1.163 | 76 | 1.5 | 2.456 | 48 | 24 | 2.714 | 29 | 24 | 2.315 | 2.171 | 192 | 1 | 4 | 1 | 1 | 4 |
| 3C | [H]/C1=C(\[H])[N+](=C([H])C([H])=C1C([H])=NO[H])C([H])([H])C([H])([H])C([H])([H])C#C[H] | 14 | 0.780 | 63 | 1 | 1.654 | 76 | 4 | 1.914 | 63 | 8 | 2.812 | 28 | 24 | 3.23 | 3.175 | 189 | 1 | 2 | 1 | 1 | 4 |
| 4C | [N-]=[N+]=NC([H])([H])OC([H])([H])[N+]=1C([H])C ([H])C([H])=C([H])C=1C([H])=NO[H] | 39 | 0.803 | 76 | 1 | 0.969 | 46 | 1 | 2.553 | 38 | 24 | 2.556 | 27 | 24 | 2.148 | 1.945 | 208 | 1 | 5 | 1 | 1 | 5 |
| 5C | [N-]=[N+]=NC([H])([H])C([H])([H])C([H])([H])C([H]) ([H])[N+]=1C([H])=C([H])C([H])=C([H])C=1C([H])=NO[H] | 18 | 2.658 | 60 | 24 | 1.021 | 73 | 1.5 | 2.408 | 38 | 24 | 1.928 | 14 | 17 | 2.957 | 2.793 | 220 | 1 | 4 | 1 | 1 | 6 |
| 6C | [H]/C1=C(/[N+](=C(/[H])C([H])=C1[H])C([H])([H])C([H])([H])C([H])([H])C#C[H])C([H])=NO[H] | 24 | 0.935 | 87 | 1 | 0.559 | 67 | 0.375 | 1.652 | 60 | 4 | 2.654 | 43 | 24 | 3.522 | 3.462 | 189 | 1 | 2 | 1 | 1 | 4 |
| 7C | [H]/C1=C(/[N+](=C(/[H])C([H])=C1[H])C([H])([H])C([H])([H])C([H])([H])C([H])([H])C#C[H])C([H])=NO[H] | 27 | 1.181 | 78 | 2 | 5.000 | 35 | 0.017 | 1.724 | 56 | 4.5 | 2.352 | 39 | 10 | 3.978 | 3.918 | 203 | 1 | 2 | 1 | 1 | 5 |
| 8C | [H]/C1=C(/[N+](=C(/[H])C([H])=C1[H])C([H])([H])C([H])([H])C#C[H])C([H])=NO[H] | 25 | 1.350 | 73 | 2 | 1.452 | 73 | 2.5 | 2.000 | 52 | 8 | 5.000 | 20 | 24 | 3.066 | 3.005 | 175 | 1 | 2 | 1 | 1 | 3 |
| 9C | [H]/C1=C(/[N+](=C(/[H])C([H])=C1[H])C([H])([H])C([H])([H])C#CC([H])([H])C#C[H])C([H])=NO[H] | 21 | 0.920 | 83 | 0.875 | 5.000 | 40 | 0.017 | 1.742 | 62 | 1.8 | 2.726 | 37 | 24 | 4.112 | 4.052 | 213 | 1 | 2 | 1 | 1 | 5 |
| 10C | [H]/C1=C(\[H])[N+](=C([H])C([H])=C1C([H])=NO[H])C([H])([H])C([H])([H])C([H])([H])C([H])([H])C#C[H] | 17 | 1.023 | 78 | 1 | 0.666 | 71 | 0.4 | 1.642 | 56 | 4.5 | 2.623 | 36 | 24 | 3.687 | 3.631 | 203 | 1 | 2 | 1 | 1 | 5 |
| 11C | [H]/C1=C(/C([H])=C([H])C([H])=[N+]1C([H])([H])C([H])([H])C([H])([H])C#C[H])C([H])=NO[H] | 15 | 0.192 | 71 | 0.17 | 0.784 | 64 | 0.5 | 0.311 | 40 | 0.2 | 2.074 | 54 | 10 | 3.23 | 3.228 | 189 | 1 | 2 | 1 | 1 | 4 |
| 12C | [N-]=[N+]=NC([H])([H])C([H])([H])C([H])([H])[N+] =1C([H])=C([H])C(=C([H])C=1[H])C([H])=NO[H] | 19 | 1.168 | 88 | 1.5 | 0.383 | 69 | 0.3 | 1.572 | 57 | 3.5 | 5.000 | 20 | 24 | 2.086 | 1.926 | 206 | 1 | 4 | 1 | 1 | 5 |
| 13C | [N-]=[N+]=NC([H])([H])C([H])([H])[N+]=1C([H]) =C([H])C(=C([H])C=1[H])C([H])=NO[H] | 14 | 1.223 | 84 | 1.5 | 0.103 | 45 | 0.2 | 1.447 | 59 | 4.7 | 2.660 | 24 | 24 | 2.023 | 1.864 | 192 | 1 | 4 | 1 | 1 | 4 |
| 14C | [H]/C1=C(/C([H])=C([H])C([H])=[N+]1C([H])([H])C([H])([H])C#C[H])C([H])=NO[H] | 13 | 1.333 | 60 | 2 | 1.620 | 63 | 4 | 0.507 | 26 | 0.6 | 5.000 | 20 | 24 | 2.774 | 2.772 | 175 | 1 | 2 | 1 | 1 | 3 |
| 15C | [H]/C1=C(\[H])[N+](=C([H])C([H])=C1C([H])=NO[H])C([H])([H])C([H])([H])C#C[H] | 11 | 0.692 | 54 | 1 | 1.724 | 70 | 7 | 2.066 | 51 | 4.5 | 2.635 | 43 | 24 | 2.774 | 2.718 | 175 | 1 | 2 | 1 | 1 | 3 |
| 16C | [H]/C1=C(/C([H])=C([H])C([H])=[N+]1C([H])([H])C([H])([H])C([H])([H])C([H])([H])C#C[H])C([H])=NO[H] | 24 | -0.335 | 64 | 0.17 | 0.291 | 66 | 0.2 | -0.002 | 38 | 0.1 | 2.088 | 60 | 13.3 | 3.687 | 3.684 | 203 | 1 | 2 | 1 | 1 | 5 |
| 17C | [H]/C1=C(\[H])[N+](=C(C([H])=C1[H])C([H])=NO[H])C([H])([H])C#C[H] | 47 | 0.531 | 65 | 0.5 | 0.407 | 71 | 0.3 | 5.000 | 20 | 24 | 5.000 | 20 | 24 | 2.744 | 2.687 | 161 | 1 | 2 | 1 | 1 | 2 |
| 1D | [H]/C(=N/O[H])C=1[N+](=NN(C=1C([H])=NO[H])C([H])([H])C2=C([H])C([H])=C([H])C([H])=C2[H])C([H])([H])[H] | 24 | 1.381 | 84 | 3 | 1.133 | 62 | 1.75 | 1.330 | 82 | 9 | 2.567 | 74 | 24 | 2.693 | 2.672 | 260 | 2 | 5 | 2 | 2 | 4 |
| 2D | [H]/C1=C(\[H])C([H])=C2/C(=C1/[H])C(=C([H])C([H])=[N+]2C([H])([H])[H])C([H])=NO[H] | 20 | 1.116 | 72 | 2 | 1.124 | 55 | 1.75 | 1.796 | 76 | 4.5 | 2.699 | 65 | 24 | 2.461 | 2.42 | 187 | 2 | 2 | 1 | 2 | 1 |
| 3D | [H]/C1=C(\[H])[N+](=C(N1C([H])([H])[H])C([H])=NO[H])C([H])([H])C([H])([H])C#C[H] | 23 | 1.243 | 81 | 2 | 0.509 | 59 | 0.33 | 1.147 | 63 | 1.5 | 5.000 | 20 | 24 | 2.243 | 2.172 | 178 | 1 | 2 | 1 | 1 | 3 |
| 4D | [N-]=[N+]=NC([H])([H])C([H])([H])C([H])([H])[N+]= 1C([H])=C([H])N(C=1C([H])=NO[H])C([H])([H])[H] | 25 | 0.583 | 85 | 0.335 | 0.355 | 77 | 0.33 | 0.786 | 62 | 1 | 2.851 | 32 | 24 | 1.555 | 1.354 | 209 | 1 | 4 | 1 | 1 | 5 |
| 5D | [N-]=[N+]=NC([H])([H])C([H])([H])C([H])([H])[N+](C ([H])([H])C([H])=NO[H])(C([H])([H])[H])C([H])([H])[H] | 16 | 2.319 | 52 | 15 | 0.943 | 69 | 1 | 1.860 | 46 | 5 | 5.000 | 20 | 24 | -0.64 | -0.75 | 186 | 0 | 4 | 1 | 0 | 6 |
| 6D | [H]C([H])(C([H])=NO[H])[N+](C([H])([H])[H])(C([H])([H])[H])C([H])([H])[H] | 21 | 3.301 | 38 | 24 | 1.391 | 71 | 2 | 2.194 | 38 | 10 | 5.000 | 20 | 24 | -1.46 | -1.47 | 117 | 0 | 2 | 1 | 0 | 2 |
| 7D | O=C(/C1=[N+](/C([H])=C([H])C([H])=C1[H])C([H])([H])[H])N([H])O[H] | 19 | 5.000 | 20 | 24 | 2.260 | 65 | 17.5 | 2.699 | 35 | 24 | 5.000 | 20 | 24 | 0.74 | 0.508 | 153 | 1 | 2 | 2 | 1 | 1 |
| 8D | O=C(C=1C([H])=C([H])[N+](=C([H])C=1[H])C([H])([H])[H])N([H])O[H] | 15 | 5.000 | 20 | 24 | 2.319 | 50 | 17.5 | 5.000 | 20 | 24 | 5.000 | 20 | 24 | 0.449 | 0.412 | 153 | 1 | 2 | 2 | 1 | 1 |
| 9D | O=C(C=1C([H])=[N+](C([H])=C([H])C=1[H])C([H])([H])[H])N([H])O[H] | 12 | 5.000 | 20 | 24 | 1.893 | 54 | 6 | 5.000 | 20 | 24 | 5.000 | 20 | 24 | 0.449 | -0.1 | 153 | 1 | 2 | 2 | 1 | 1 |
| 10D | [H]/C(=N/O[H])C=1N=NN(C=1C([H])=NO[H])C([H])([H])C2=C([H])C([H])=C([H])C([H])=C2[H] | 12 | 2.678 | 34 | 24 | 2.060 | 47 | 6.5 | 2.432 | 36 | 24 | 5.000 | 20 | 24 | 1.969 | 1.961 | 245 | 2 | 6 | 2 | 2 | 4 |
| 11D | [H]C1([H])C(=NO[H])C([H])([H])[S+](C1([H])[H])C([H])([H])[H] | 14 | 5.000 | 20 | 24 | 2.252 | 23 | 17 | 5.000 | 20 | 24 | 5.000 | 20 | 24 | 0.48 | 0.477 | 132 | 0 | 2 | 1 | 1 | 0 |
| 12D | [H]C=1[S]C(=NC=1C([H])([H])[H])C([H])=NO[H] | 14 | 5.000 | 20 | 24 | 1.767 | 60 | 4 | 2.553 | 38 | 24 | 5.000 | 20 | 24 | 0.801 | 0.655 | 142 | 1 | 3 | 1 | 1 | 1 |
| 13D | [H]C([H])(C([H])=NO[H])[N+](C([H])([H])[H])(C([H])([H])C([H])([H])C#C[H])C([H])([H])[H] | 18 | 2.569 | 68 | 24 | 0.888 | 70 | 1 | 1.684 | 30 | 2.1 | 5.000 | 20 | 24 | 0.049 | 0.045 | 155 | 0 | 2 | 1 | 0 | 4 |
| 14D | [H]/C1=C(/C([H])=C([H])[S+]1C([H])([H])[H])C([H])=NO[H] | 13 | 5.000 | 20 | 24 | 5.000 | 20 | 24 | 5.000 | 20 | 24 | 5.000 | 20 | 24 | 1.42 | 1.417 | 142 | 1 | 2 | 1 | 1 | 1 |
| 15D | [H]C=1N=C([S]C=1[H])C(=NO[H])C([H])([H])[H] | 15 | 2.538 | 40 | 24 | 0.887 | 27 | 1 | 5.000 | 20 | 24 | 5.000 | 20 | 24 | 0.499 | 0.498 | 142 | 1 | 3 | 1 | 1 | 1 |
| 16D | [H]C=1[N+](=C(N(C=1[H])C([H])([H])[H])C([H])=NO[H])C([H])([H])[H] | 15 | 1.308 | 83 | 3 | 1.177 | 66 | 1.5 | 1.618 | 65 | 4 | 5.000 | 20 | 24 | 0.73 | 0.657 | 140 | 1 | 2 | 1 | 1 | 1 |
| 17D | [F]C=1C([F])=C([F])C([F])=C(C=1[F])C([H])=NO[H] | 23 | 5.000 | 20 | 24 | 2.276 | 70 | 24 | 2.481 | 22 | 5 | 5.000 | 20 | 24 | 2.684 | 2.683 | 211 | 1 | 2 | 1 | 1 | 1 |
| 18D | [H]/C1=C(/N=C(C([H])=C1[H])C([H])=NO[H])C([H])=NO[H] | 16 | 5.000 | 20 | 24 | 1.866 | 39 | 5 | 5.000 | 20 | 24 | 5.000 | 20 | 24 | 1.189 | 1.234 | 165 | 1 | 5 | 2 | 1 | 2 |
| 19D | [H]/C1=C(/[S+](C([H])=C1[H])C([H])([H])[H])C([H])=NO[H] | 14 | 5.000 | 20 | 24 | 5.000 | 20 | 24 | 5.000 | 20 | 24 | 5.000 | 20 | 24 | 1.712 | 1.712 | 142 | 1 | 2 | 1 | 1 | 1 |
| 20D | [H]C1([H])[S]C([H])([H])C([H])([H])C1=NO[H] | 32 | 5.000 | 20 | 24 | 2.060 | 63 | 10 | 5.000 | 20 | 24 | 5.000 | 20 | 24 | 0.378 | 0.378 | 117 | 0 | 3 | 1 | 1 | 0 |
| 21D | [H]C=1N=C(N(C=1[H])C([H])([H])[H])C([H])=NO[H] | 22 | 2.367 | 71 | 24 | 1.090 | 43 | 0.625 | 1.684 | 38 | 3.5 | 5.000 | 20 | 24 | 0.156 | 0.266 | 125 | 1 | 3 | 1 | 1 | 1 |
| 22D | [H]/C1=C(\[H])C([H])=C2/C(=C1/[H])C(=C([H])N2[H])C([H])=NO[H] | 22 | 5.000 | 20 | 24 | 5.000 | 20 | 24 | 5.000 | 20 | 24 | 5.000 | 20 | 24 | 1.95 | 1.949 | 160 | 2 | 2 | 2 | 2 | 1 |
| 23D | [H]C=1N=C([S]C=1[H])C([H])=NO[H] | 16 | 2.569 | 55 | 24 | 1.133 | 59 | 1.75 | 2.387 | 42 | 24 | 5.000 | 20 | 24 | 0.518 | 0.517 | 128 | 1 | 3 | 1 | 1 | 1 |
| 24D | [H]C=1[S]C([H])=C([H])C=1C([H])=NO[H] | 14 | 5.000 | 20 | 22 | 2.602 | 25 | 17.5 | 5.000 | 20 | 24 | 5.000 | 20 | 24 | 1.318 | 1.317 | 127 | 1 | 2 | 1 | 1 | 1 |
| 25D | [H]/C1=C(/[S]C([H])=C1[H])C([H])=NO[H] | 8 | 5.000 | 20 | 12 | 2.553 | 43 | 24 | 5.000 | 20 | 24 | 5.000 | 20 | 24 | 1.61 | 1.609 | 127 | 1 | 2 | 1 | 1 | 1 |
| 26D | [H]/C1=C(\[H])C([H])=C2/C(=C1/[H])C(=C([H])N2C([H])([H])[H])C([H])=NO[H] | 25 | 5.000 | 20 | 24 | 5.000 | 20 | 24 | 5.000 | 20 | 24 | 5.000 | 20 | 24 | 2.156 | 2.154 | 174 | 2 | 2 | 1 | 2 | 1 |
| 27D | [H]C=1N(N=NC=1C([H])=NO[H])C([H])([H])C2=C([H])C([H])=C([H])C([H])=C2[H] | 16 | 5.000 | 20 | 24 | 2.125 | 74 | 11 | 2.824 | 48 | 24 | 5.000 | 20 | 24 | 1.851 | 1.847 | 202 | 2 | 4 | 1 | 2 | 3 |
| 2-PAM | [H]C=1C([H])=[N+](/C(=C(/[H])C=1[H])C([H])=NO[H])C([H])([H])[H] | 8 | 0.872 | 80 | 1.357 | 1.054 | 72 | 1 | 1.249 | 56 | 1.7 | 2.670 | 27 | 12 | 1.552 | 1.45 | 137 | 1 | 2 | 1 | 1 | 1 |
| HI-6 | O=C(/C1=C(\[H])C([H])=[N+](C([H])=C1[H])C([H])([H])OC([H])([H])[N+]2=C([H])C([H])=C([H])C([H])=C2C([H])=NO[H])N([H])[H] | 11 | 0.959 | 92 | 0.6667 | -0.050 | 71 | 0.17 | 1.418 | 62 | 1.8 | 2.194 | 14 | 5 | 1.641 | 1.373 | 288 | 2 | 4 | 2 | 2 | 6 |
| Obid­oxime | [H]/C1=[N+](/C([H])=C([H])C(=C1[H])C([H])=NO[H])C([H])([H])OC([H])([H])[N+]2=C([H])C([H])=C(C([H])=C2[H])C([H])=NO[H] | 14 | -0.015 | 61 | 0.4175 | 0.402 | 66 | 0.833 | 1.510 | 54 | 3 | 2.003 | 40 | 4 | 2.174 | 2.169 | 288 | 2 | 5 | 2 | 2 | 6 |
| TMB-4 | [H]/C1=C(/C([H])=C([H])[N+](=C1[H])C([H])([H])C([H])([H])C([H])([H])[N+]2=C([H])C([H])=C(C([H])=C2[H])C([H])=NO[H])C([H])=NO[H] | 12 | 0.431 | 65 | 0.335 | 1.217 | 85 | 2 | 1.161 | 43 | 2 | 2.258 | 62 | 6 | 2.403 | 2.397 | 286 | 2 | 4 | 2 | 2 | 6 |

**Table S2**. Individual principal component (PC) analysis on 115 compounds tested for reactivation of GB-inhibited BChE. List of loadings for 18 variables ordered according to PC1. Proportion of variance is listed in the brackets with the cumulative proportion of variance for the selected components 82.2 %.

|  | Loading | | |
| --- | --- | --- | --- |
|  | PC1 (51.8 %) | PC2 (19.3 %) | PC3 (11.1 %) |
| Eigenvalue | 9.32 | 3.52 | 2.0 |
| Time, h (GB) | 0.627 | -0.239 | -0.634 |
| - log (*k*_obs_ min^-1^) (GB) | 0.566 | -0.172 | -0.755 |
| Molecular fractional polar surface area | 0.252 | -0.527 | -0.303 |
| ADMET Alog*P*98 | -0.244 | 0.916 | -0.226 |
| Alog*P* | -0.429 | 0.856 | -0.154 |
| log*D* | -0.433 | 0.855 | -0.159 |
| Dipole magnitude | -0.505 | 0.190 | -0.107 |
| Oxime inhibition, | -0.578 | 0.173 | -0.293 |
| Number of H bond donor atoms | -0.614 | -0.574 | -0.019 |
| React_max_, % (GB) | -0.642 | 0.044 | 0.671 |
| Number of H bond acceptor atoms | -0.847 | -0.398 | -0.147 |
| ADMET PSA 2D | -0.868 | -0.434 | -0.071 |
| Number of rings | -0.882 | 0.049 | -0.362 |
| Number of aromatic rings | -0.893 | 0.061 | -0.334 |
| Number of rotational bonds | -0.932 | -0.159 | 0.078 |
| Molecular surface area | -0.964 | -0.186 | -0.014 |
| Molecular volume | -0.970 | -0.139 | -0.058 |
| Molecular weight | -0.974 | -0.139 | -0.110 |

**Table S3**. Individual principal component (PC) analysis on 115 compounds tested for reactivation of GF-inhibited BChE. List of loadings for 18 variables ordered according to PC1. Proportion of variance is listed in the brackets with the cumulative proportion of variance for the selected components 79.3 %.

|  | Loadings | | |
| --- | --- | --- | --- |
|  | PC1 (49.9 %) | PC2 (19.7 %) | PC3 (9.7 %) |
| Eigenvalue | 8.99 | 3.54 | 1.75 |
| - log (*k*_obs_ min^-1^) (GF) | 0.557 | 0.275 | -0.622 |
| Time, h (GF) | 0.446 | 0.209 | -0.680 |
| Molecular fractional polar surface area | 0.202 | -0.492 | -0.353 |
| ADMET Alog*P*98 | -0.198 | 0.948 | 0.020 |
| Alog*P* | -0.377 | 0.884 | 0.034 |
| log*D* | -0.381 | 0.885 | 0.025 |
| React_max_,% (GF) | -0.439 | -0.060 | 0.695 |
| Dipole magnitude | -0.505 | 0.235 | 0.064 |
| Oxime inhibition, % | -0.590 | 0.247 | -0.076 |
| Number of H bond donor atoms | -0.641 | -0.516 | -0.195 |
| Number of H bond acceptor atoms | -0.876 | -0.315 | -0.178 |
| Number of rings | -0.890 | 0.174 | -0.301 |
| ADMET PSA 2D | -0.890 | -0.363 | -0.158 |
| Number of aromatic rings | -0.898 | 0.182 | -0.279 |
| Number of rotational bonds | -0.936 | -0.115 | 0.076 |
| Molecular surface area | -0.973 | -0.119 | -0.021 |
| Molecular volume | -0.978 | -0.064 | -0.051 |
| Molecular weight | -0.983 | -0.054 | -0.104 |

**Table S4**. Individual principal component (PC) analysis on 115 compounds tested for reactivation of VX-inhibited BChE. List of loadings for 18 variables ordered according to PC1. Proportion of variance is listed in the brackets with the cumulative proportion of variance for the selected components 82.1 %

|  | Loading | | |
| --- | --- | --- | --- |
|  | PC1 (50.3 %) | PC2 (19.3 %) | PC3 (12.5 %) |
| Eigenvalue | 11.46 | 4.18 | 3.82 |
| Time, h (VX) | 0.487 | 0.152 | -0.797 |
| - log (*k*_obs_ min^-1^) (VX) | 0.481 | 0.120 | -0.793 |
| Molecular fractional polar surface area | 0.242 | -0.465 | -0.512 |
| ADMET AlogP98 | -0.206 | 0.950 | -0.014 |
| Alog*P* | -0.390 | 0.884 | 0.022 |
| log*D* | -0.394 | 0.883 | 0.022 |
| Dipole magnitude | -0.487 | 0.240 | -0.143 |
| Oxime inhibition, | -0.567 | 0.258 | -0.255 |
| React_max_, % (VX) | -0.590 | -0.110 | 0.612 |
| Number of H bond donor atoms | -0.642 | -0.536 | -0.107 |
| Number of H bond acceptor atoms | -0.859 | -0.321 | -0.243 |
| ADMET PSA 2D | -0.883 | -0.374 | -0.172 |
| Number of rings | -0.888 | 0.157 | -0.291 |
| Number of aromatic rings | -0.898 | 0.163 | -0.261 |
| Number of rotational bonds | -0.936 | -0.129 | 0.048 |
| Molecular surface area | -0.973 | -0.136 | -0.027 |
| Molecular volume | -0.979 | -0.082 | -0.053 |
| Molecular weight | -0.983 | -0.072 | -0.102 |

**Table S5**. Individual principal component (PC) analysis on 115 compounds tested for reactivation of GA-inhibited BChE. List of loadings for 18 variables ordered according to PC1. Proportion of variance is listed in the brackets with the cumulative proportion of variance for the selected components 78.5 %.

|  | Loadings | | |
| --- | --- | --- | --- |
|  | PC1 (47.0%) | PC2 (21.4%) | PC3 (10.1%) |
| Eigenvalue | 8.46 | 3.85 | 1.83 |
| Molecular fractional polar surface area | 0.210 | -0.512 | 0.002 |
| - log (*k*_obs_ min^-1^) (GA) | 0.188 | -0.490 | -0.729 |
| Time, h (GA) | 0.001 | -0.459 | -0.521 |
| React_max_, % (GA) | -0.139 | 0.493 | 0.669 |
| ADMET Alog*P*98 | -0.249 | 0.886 | -0.313 |
| Alog*P* | -0.422 | 0.836 | -0.242 |
| log*D* | -0.427 | 0.835 | -0.245 |
| Dipole magnitude | -0.516 | 0.236 | 0.112 |
| Oxime inhibition, % | -0.595 | 0.090 | -0.488 |
| Number of H bond donor atoms | -0.626 | -0.524 | 0.204 |
| Number of H bond acceptor atoms | -0.865 | -0.370 | 0.051 |
| ADMET PSA 2D | -0.877 | -0.398 | 0.124 |
| Number of rotational bonds | -0.919 | -0.132 | 0.105 |
| Number of rings | -0.922 | 0.061 | -0.129 |
| Number of aromatic rings | -0.928 | 0.073 | -0.119 |
| Molecular surface area | -0.964 | -0.161 | 0.071 |
| Molecular volume | -0.976 | -0.113 | 0.050 |
| Molecular weight | -0.985 | -0.116 | 0.022 |

**Figure S2.** Plot of physicochemical properties of the studied oximes (N) (Table S1). Values below the red line represent recommended values for the CNS-active drugs [1]

Reference

[1] H. Pajouhesh, G.R. Lenz. Medicinal Chemical Properties of Successful Central Nervous System Drugs. *NeuroRX* **2** (2005) 541-553, <https://doi.org/10.1602/neurorx.2.4.541>
